# Quantifying the relationship between cell proliferation and morphology during development of the face

**DOI:** 10.1242/dev.204511

**Published:** 2025-04-07

**Authors:** Lucas D. Lo Vercio, Rebecca M. Green, Andreas Dauter, Elizabeth C. Barretto, Marta Vidal-García, Jay Devine, Marta Marchini, Samuel Robertson, Xiang Zhao, Anandita Mahika, M. Bilal Shakir, Sienna Guo, Julia C. Boughner, Heather Szabo-Rogers, Wendy Dean, Arthur D. Lander, Ralph S. Marcucio, Nils D. Forkert, Benedikt Hallgrímsson

**Affiliations:** ^1^ Department of Cell Biology & Anatomy, Cumming School of Medicine, University of Calgary, Calgary, AB T2N 4N1, Canada; ^2^ Alberta Children's Hospital Research Institute, University of Calgary, Calgary, AB T2N 4N1, Canada; ^3^ McCaig Bone and Joint Institute, University of Calgary, Calgary, AB T2N 4N1, Canada; ^4^ Department of Oral and Craniofacial Sciences, Center for Craniofacial and Dental Genetics, University of Pittsburgh, Pittsburgh, PA 15219, USA; ^5^ Medical Imaging Research Center, MIRC, UZ Leuven, B-3000 Leuven, Belgium; ^6^ Department of Anatomy, Physiology and Pharmacology, College of Medicine, University of Saskatchewan, Saskatoon, SK S7N 5E5, Canada; ^7^ Department of Developmental and Cell Biology, University of California, Irvine, Irvine, CA 92697, USA; ^8^ Department of Orthopaedic Surgery, University of California San Francisco, San Francisco, CA 94110, USA

**Keywords:** Light-sheet imaging, Image segmentation, Convolutional neural networks, Mouse embryo, Developmental biology, Morphometrics

## Abstract

Morphogenesis requires highly coordinated, complex interactions between cellular processes: proliferation, migration and apoptosis, along with physical tissue interactions. How these cellular and tissue dynamics drive morphogenesis remains elusive. Three dimensional (3D) microscopic imaging holds great promise, and generates elegant images, but generating even moderate throughput for quantified images is challenging for many reasons. As a result, the association between morphogenesis and cellular processes in 3D developing tissues has not been fully explored. To address this gap, we have developed an imaging and image analysis pipeline to enable 3D quantification of cellular dynamics along with 3D morphology for the same individual embryo. Specifically, we focus on how 3D distribution of proliferation relates to morphogenesis during mouse facial development. Our method involves imaging with light-sheet microscopy, automated segmentation of cells and tissues using machine learning-based tools, and quantification of external morphology by geometric morphometrics. Applying this framework, we show that changes in proliferation are tightly correlated with changes in morphology over the course of facial morphogenesis. These analyses illustrate the potential of this pipeline to investigate mechanistic relationships between cellular dynamics and morphogenesis during embryonic development.

## Teaser

**Summary:** Demonstration of a method for quantifying 3D proliferation staining from light-sheet microscopy data, directly relating proliferation patterns to morphology over a developmental time series in the mouse embryo and showing that this method is able to separate mutant and wild-type embryos.

## INTRODUCTION

Disentangling the mechanisms of morphogenesis that translate the behavior of individual cells to an intricate three-dimensional (3D) organismal form has been a major objective of embryology and developmental biology since the early 19th century. The cellular basis for morphogenesis is complex, involving spatiotemporal variation in cell proliferation, apoptosis, adhesion and polarity. Morphogenesis also involves interactions between cells, the extracellular matrix, and mechanical forces that emanate from surrounding tissues and the extra-embryonic environment (Boehm et al., 2010; Seilacher, 1991; Newman and Comper, 1990; Niessen et al., 2011; Davies, 2013). Connecting these mechanisms to 3D change in organismal form requires quantification of cellular processes in whole embryonic structures over developmental time. While three dimensional morphology can be quantified from computed microtomography and optical projection tomography (OPT) images (Parsons et al., 2008; Boughner et al., 2008; Xu et al., 2015; Martínez-Abadías et al., 2018), cellular dynamics are much less accessible in three dimensions. This is because the vast majority of quantification of cellular dynamics is based on analysis of serial histological sections, with localized sampling rather than whole embryonic structures (Ramaesh and Bard, 2003; Miklius and Hilgenfeldt, 2011; Russ and Dehoff, 2012). Serial histological sections are rife with artifacts ranging from distortions from fixation and sectioning (Xiao et al., 2010) to plane of section artifacts that occur when complex 3D structures are reduced to two dimensions (Russ and Dehoff, 2012). Serial sectioning of whole embryonic structures is also labor and time intensive, which makes this method unsuitable for analysis of large samples. However, recent advances in 3D imaging of whole tissue samples, including entire embryos, as well the ability to combine such images with multiple molecular markers is now creating the opportunity for true quantitative integration of cellular dynamics and morphology. These imaging modalities also generate large and complex datasets that demand novel image processing and analysis methods. In this study, we deploy a novel imaging and image analysis pipeline to examine cell proliferation in the developing face, thereby advancing the capacity for quantitative integration of cellular dynamics and morphology.

The development of the vertebrate face involves directional outgrowth and fusion of distinct facial prominences that form different facial regions. In mammals, the upper jaw forms from divisions of the frontonasal prominence along the midline – the medial and lateral nasal prominences – fusing together and with the maxillary prominence (Chai and Maxson, 2006). The facial prominences must grow in a coordinated manner that includes both outgrowth and alignment to allow for fusion (Chai and Maxson, 2006; Green et al., 2015). Although the processes involved in outgrowth of structures such as limb buds have been fairly well characterized (Boehm et al., 2010), these studies have been more difficult to perform in the face due to the complexity of signaling pathway interactions and morphology. Unlike the limb, outgrowth of the facial prominences is orchestrated by multiple signaling centers, including around the frontonasal prominence (WNT, FGFs), in the leading edge of the maxilla (FGFs, BMPs) and in the forebrain (SHH, BMPs) (Marchini et al., 2021). These signals drive a combination of local cell proliferation, migration of neural crest cells, and additionally likely affect mechanical properties of the tissue. The complex interplay of growth, cell migration and signaling has made it difficult to tease apart the roles of individual factors. Although there is a general intuition that spatiotemporal regulation of cell proliferation within the facial prominences is important in face formation, this regulation has not been shown quantitatively. Further, the role of proliferation in relation to other potential processes, such as apoptosis, adhesion, polarity, or mechanical influences from the epithelium, needs to be understood in much finer detail to explain facial development and account for congenital facial malformations, such as orofacial clefts.

Recent advances in tissue-clearing methods coupled with light-sheet fluorescence microscopy (LSFM) allow visualization of individual cells within whole embryos or anatomical structures (Weber et al., 2014; Yue et al., 2020; Udan et al., 2014; Elisa et al., 2018). This visualization enables quantification of cellular level variation as well as the morphology of the developing tissues at the level of individual embryos (McDole et al., 2018; Hobson et al., 2022). Accordingly, cellular dynamics, such as proliferation, orientation, apoptosis, cell size and cell density, can be related quantitatively to variation in morphology. Importantly, these analyses can be performed on individuals, as opposed to group-wise, allowing for cellular-level investigations of the mechanisms underlying among-individual variation in morphology. This individual-level investigation is necessary for elucidating mechanisms for genetically complex malformations or those which have variable penetrance or expressivity (Hallgrimsson et al., 2019). However, even with light-sheet microscopy, this task is challenging. In mice, for example, tissue density precludes single-cell visualization without clearing to allow light penetration after embryonic day (E) 8.5 (McDole et al., 2018). Quantifying variation among embryos requires some form of image registration to identify homology between corresponding anatomical locations. However, the large amount of shape changes characteristic of morphogenesis complicates the construction of atlas-based registration pipelines without copious quantities of data (Wong et al., 2015). LSFM images are prone to artifacts from optical aberrations or deviations in refractive index across a specimen. These issues present challenges for developing segmentation protocols, either human or computational, as a region of an image may be out of focus. Further, the images files are large, ranging from 300 MB to 1 TB, depending on embryo size and image resolution. Therefore, LSFM images require extensive computational resources for data management and analysis. Overcoming these obstacles has been challenging with available tools and conventional methods.

As such, previous work towards this goal has been hampered by both limitations in imaging technology as well as image processing and informatics. OPT has been used to create anatomical images and quantitative analyses for gene expression (Sharpe et al., 2002) as well as markers of cellular dynamics (Boehm et al., 2010). The limitation here is that, as the anatomical structures to be imaged get larger, the effective resolution of OPT imaging decreases, as this is ultimately dependent on the geometry and detection aperture (Wong et al., 2013; Liu et al., 2019). While early zebrafish embryos are sufficiently small to allow such imaging (Lindsey and Kaslin, 2017), structures such as whole mouse embryo heads larger than about E10.5 are too large to allow cellular level imaging with currently available OPT systems. Light-sheet microscopy overcomes this limitation of OPT using tiling and laser optics by illuminating a thin sheet of the sample (Olarte et al., 2018). This method also reduces photodamage, which can occur in confocal fluorescence microscopy (Olarte et al., 2018). The image processing and imaging informatics gap is that all of these methods generate large and noisy volumetric image sets that require extensive post-processing and advanced registration methods. Our work addresses this gap using advanced image processing methods.

In a previous paper, we (Lo Vercio et al., 2022) demonstrated that convolutional neural networks (CNNs), particularly U-net architectures, can efficiently segment the mesenchyme (Mes) and neural ectoderm (NE) in nuclear-stained 2D LSFM images. This type of deep learning architecture has also been used for other aspects of light-sheet data processing (Yin et al., 2022; Hallou et al., 2021). Furthermore, CNNs efficiently segmented cells labeled for a proliferation marker in LSFM images of E9.5-E10.5 embryos. Here, we leverage this advanced image processing approach to develop and apply a method to quantify the relationship between cellular dynamics and 3D morphology in embryonic tissues based on light-sheet microscopy. We first apply our previously developed tools for segmenting tissues and cells at the individual level (Lo Vercio et al., 2022). Next, a 3D image registration pipeline technique (Devine et al., 2020) is employed to create 3D atlases that separate neural and non-neural tissues and contain volumetric representations of cell proliferation. We then apply the tools of geometric morphometrics to quantify spatiotemporal variation in cell proliferation along with embryonic surface morphology. Specifically, we seek to determine (1) the relationship between proliferation and morphogenesis, and (2) the contribution of anatomical variation in proliferation to among-individual variation in cranial morphology. Successful quantitative integration of cellular dynamics is important to unravel the mechanisms of morphogenesis, but also the mechanistic basis for among-individual variation, including the etiology of structural birth defects.

## RESULTS

### Light-sheet imaging and workflow to build atlases of shape, tissues and cellular dynamics from mouse embryos

**Fig. 1.**
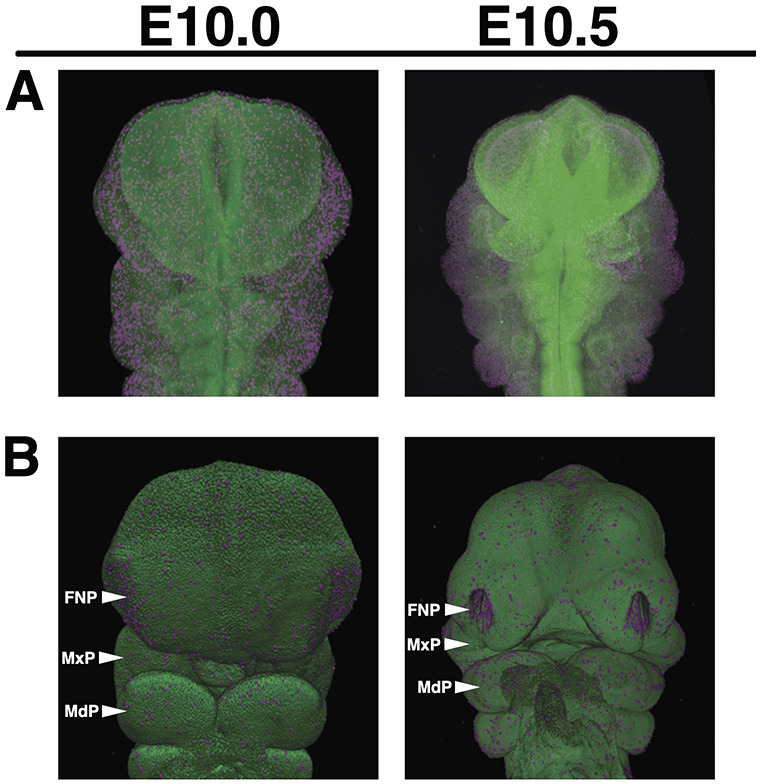
**Lightsheet visualization of mouse facial proliferation.** (A) Maximum projection intensity images in which Nuclear Green has been used to stain nuclei; phospho-Histone H3 (pHH3) has been stained in magenta to mark cell proliferation. Embryos were harvested at E10-E11 from C57Bl/6J mice following timed matings. (B) Space-filling representation of the same embryos from A. Embryos were cleared and prepared following the CUBIC protocol, immunostained, and mounted in 1.5% low-melt agarose. Images were captured on a Zeiss Lightsheet Z1 microscope and analyzed in Arivis. Embryo staging was established by counting tail somites. Representative images of *n*=22 samples. Images are not to scale. FNP, frontonasal prominence; MdP, mandibular prominence; MxP, maxillary prominence. See Movies 1 and 2 for movie representations of the raw data.

**Fig. 2.**
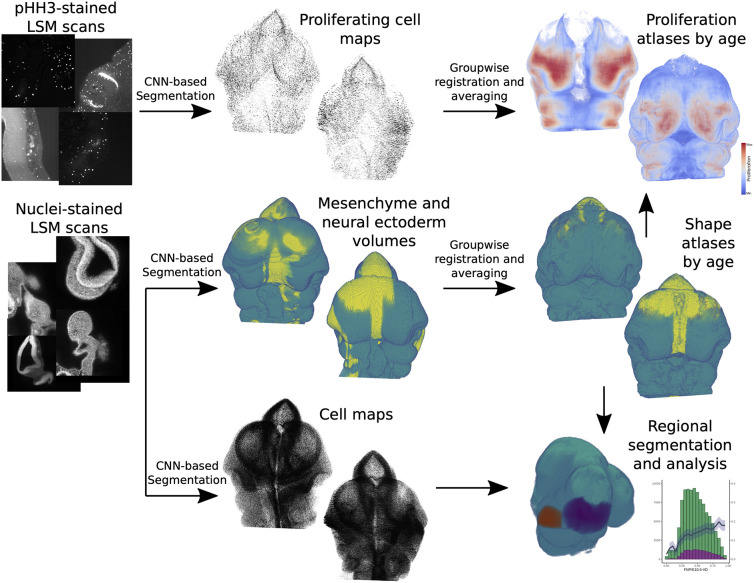
**Overview of the workflow for creating shape and proliferation atlases from LSFM scans.** Twenty mouse embryos are stained for nuclei (Nuclear Green) and proliferating cells (pHH3). For each specimen, proliferating cell maps and tissues are automatically segmented using CNNs (mesenchyme in teal, neural ectoderm in yellow). For each age group (E10, E10.5, E11 and E11.5), groupwise affine-registration is performed using the tissue segmentation volume. Then, the resulting transformation for each sample is applied to its proliferating cell map. The shape atlas per age is obtained by majority voting in each voxel. The proliferation atlases are produced by smoothing and normalizing each proliferating cell map, and then averaging the resulting heat maps of the age group (minimum proliferation in blue, maximum proliferation in red).

LSFM imaging of mouse facial tissue can be used to generate both cellular level volumetric data and a quantifiable 3D exterior surface (Fig. 1). Building on previous work (Lo Vercio et al., 2022), we established an automatic workflow for segmenting, registering and quantifying cellular dynamics from LSFM scans of mouse embryos (Fig. 2). Here, we scanned 20 wild-type C57BL/6J mouse embryos ranging in age from E10 to E11.5. This age range encompasses early facial morphogenesis and captures the cell dynamics involved in facial prominence outgrowth. The embryos were separated into half-day groups based on tail somite number, with five embryos per group. Half-day intervals were originally chosen to compose well-matched groups that can be used for atlas generation. We produced registered volumetric image sets from these scans and analyzed spatiotemporal patterning of proliferation in whole embryonic heads.

An LSFM instrument generates data as a stack of 2D slices. Each slice is generated as the laser sheet passes through the sample. The within-slice resolution is typically six to eight times higher than the between-slice resolution. Here, our analysis focuses on using the stack data: the tissues, total cells and proliferating cells are segmented in the 2D images, then the anisotropic *z*-stacks of segmented images are converted to isotropic volumes (Fig. 2, middle column). A group-wise registration strategy based on the tissue segmentation can be used to create atlases of shapes and cellular dynamics (Fig. 2, right). This pipeline and accompanying documentation is available from https://github.com/lucaslovercio/LSMprocessing.

### Shape, tissue and proliferation atlases between E10.0 and E11.5

**Fig. 3.**
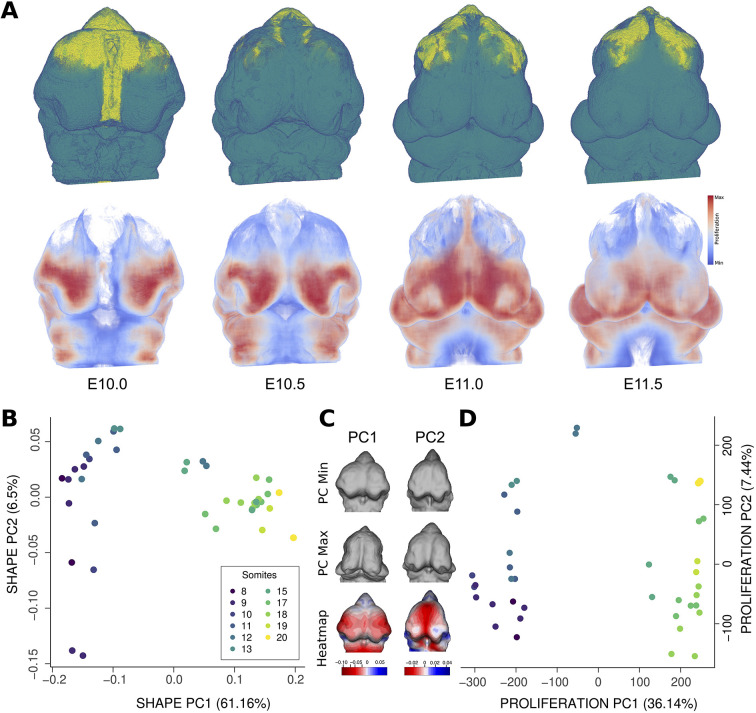
**Tissues, proliferation and shape distribution of the dataset.** (A) Tissue and proliferation atlases for C57Bl/6J embryos between E10.0 and E11.5 (*n*=20 samples; left and right sides were mirrored and analyzed independently). The top row presents the tissue atlases for embryonic ages E10.0, E10.5, E11.0 and E11.5, showing mesenchyme in teal and neural ectoderm in yellow. Voxels are displayed as slightly transparent for visualization purposes. The bottom row shows the proliferation map atlases for the same embryonic ages, only in the mesenchyme. (B,D) Principal component analysis of Procrustes shape coordinates (C) and facial proliferation (D) for the whole embryonic dataset. Specimens are colored by tail somite count, as a proxy for age. Dataset comprises embryos from 8 to 20 somites. (C) Representative shapes moving across PC1 and PC2.

An atlas of neural and non-neural tissues was generated for each age group. Images of these atlases are shown in Fig. 3A, top row. Our analysis focused on the non-neural tissues, primarily mesenchyme, as most of the key events happening during facial morphogenesis happen in mesenchymal tissues. Furthermore, the dense neural tissue along with image artifacts, such as blurriness and signal loss, hinder the proper segmentation of the tissue itself and its cells. From here, mean proliferation was calculated for each group of specimens (Fig. 3A, bottom row). In general, we observed high levels of proliferation in the frontonasal, mandibular and maxillary prominences – areas where shape change is happening at these points in development.

In order to better understand the change in morphology and proliferation over the age series, we used landmark-based geometric morphometrics. We used 37 landmarks that are focused on the front of the face to capture the morphological shifts in the facial prominences (Fig. 3C) (Percival et al., 2014). To understand how the overall morphology of the embryo changes with time, we used principal component analysis (PCA) to identify the largest axes of variance in our dataset (Fig. 3B). For the PCA of facial shape coordinates, PC1 explained most of the variance (61.6%) and PC2 only 6.5% of the overall variance. Shape changes on PC1 were directly correlated to somite count, strongly separating samples with 8-14 somites (left region of the morphospace along PC1, negative values) from samples with 15-20 somites (right region of the morphospace along PC1, positive values). Interestingly, although we attempted to group the embryos using tail somite number, they still clustered morphologically into two distinct groups, E10-E10.5 and E11-E11.5. This clustering effect seems to be strongly driven by changes in the nasal prominence region. We found similar patterns on the PCA of proliferation in the front of the face, in which PC1 explained 36.14% of the overall variance, while PC2 only explained an additional 7.44% (Fig. 3D). We also found a strong separation of the same two age groups we observed in the shape PCA: samples with 8-14 somites (younger embryos) and samples with 15-20 somites (older embryos).

**Fig. 4.**
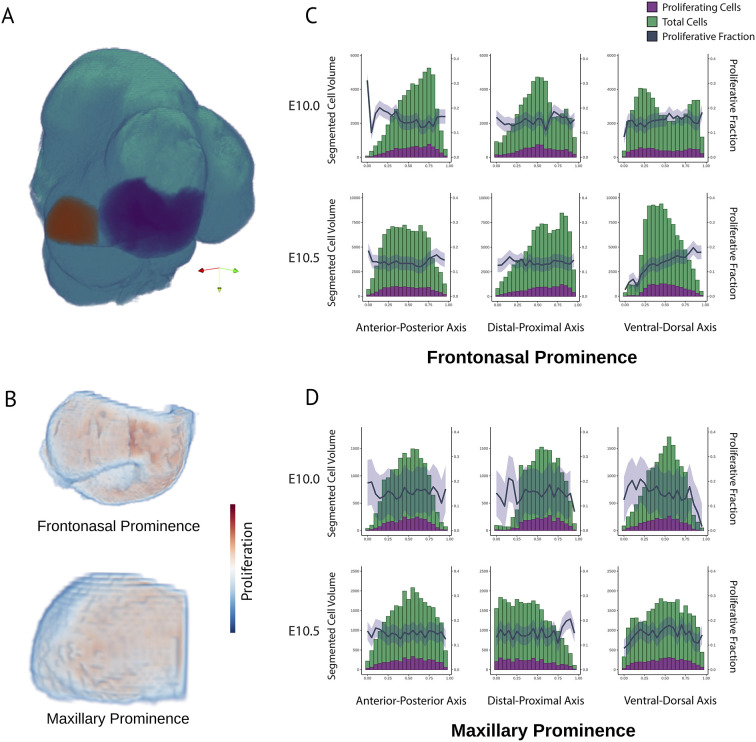
**Segmented cell volume and proliferative fraction along each tissue axis at E10.0 and E10.5 in key tissues.** (A) Segmentation of the maxillary prominence (orange) and frontonasal prominence (purple) on the average tissue atlas. Colored arrows denote anatomical directions: posterior (red), anterior (green), inferior (yellow). (B) Proliferative heatmaps within segmented tissues in the same view as in A. (C,D) Cell volumes and proliferative cell volumes are compared along each axis and reveal largely homogeneous proliferation in these averaged volumes. The blue line represents the proliferative fraction, or the proportion of proliferating nuclei to total nuclei, and the blue shaded area represents the s.e.m. between specimens. *n*=5 per age group.

This observation raised the question of whether proliferation was uniform across these regions. We hypothesized that differences in proliferation density across developing facial prominences precede morphological change. To test this hypothesis, we manually segmented the frontonasal and maxillary prominences at the earlier time points (E10-E10.5) and calculated rates of proliferation across each developmental axis: anterior to posterior, dorsal to ventral, and distal to proximal. These tissues are highly proliferative and commonly perturbed in development, making them important morphological targets (Elmsie and Reardon, 1998). We calculated the absolute and proportional volume of proliferating nuclei compared to total nuclei across each axis of a prominence, averaging across all specimens in each group (Fig. 4). We found that the proportion of proliferating cells remains stable across most biological axes in these prominences. Additionally, there were no significant differences in mean proliferative fraction within each prominence, suggesting that the rate of proliferation remains similar in actively proliferating facial tissues. Tissue-specific means were similar, ranging between 14% and 18% of cells proliferating. We did observe a difference in the E10.5 frontonasal prominence along the dorsal-ventral axis, where cells proliferated more densely towards the dorsal side (interior) of the tissue (*P*<0.0001). Notably, apparent patterns in proliferation may be related to the overall density of cells across tissues, which changed with time and prominence location. These apparent differences in cell densities could relate to morphological change over development and will need to be more carefully examined in future studies.

### Correlation of morphology and proliferation

**Fig. 5.**
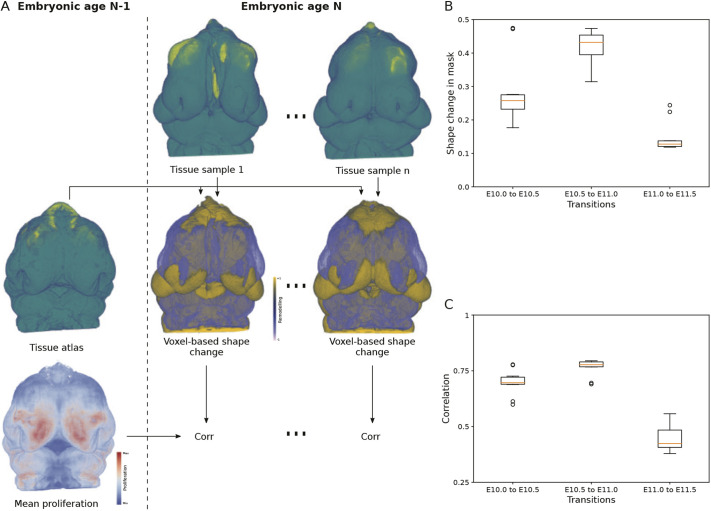
**Voxel-based analysis of relation between proliferation and shape change in the face.** (A) Workflow to relate the shape and proliferation atlases of the previous embryonic age N−1 to the shape of each of the samples belonging to the present embryonic age N. All embryos were subject to Procrustes superimposition to remove size difference (not shown). A differential volume (−1, 0, +1) for each sample at age N is obtained by comparing to the mesenchyme of the age N−1 atlas (middle row; −1 in purple, +1 in gold; *n*=15 samples). Then, each voxel of the differential volume at age N was associated with the corresponding voxel of the proliferation atlas of age N−1. Finally, Pearson's linear correlation coefficient is computed only in a mask corresponding to the mesenchyme of the face. (B) Following size removal, box plots showing the proportion of voxels in the fixed mask of the face that presents outward (+1) or inward (−1) shape change between the consecutive ages studied in this work (E10.0-E11.5). (C) Box plots presenting the absolute value of the correlation coefficient between the proliferation at age N−1 with the shape change from N−1 to N, in consecutive ages. Box plots represent a standard box plot, where the middle line (red) represents the median, the box represents the 1st-3rd interquartile range, the whiskers represent 1.5× the interquartile range and dots represent outlying data points.

Spatiotemporal patterning of cell proliferation has long been thought to be a key driver of morphogenesis (Francis-West et al., 1998; Marcucio et al., 2015). To determine whether the spatial patterning of proliferation is correlated with morphological change, we used voxel-based methods to identify between-group differences and to examine the correlations between shape (morphology) change and proliferation during the phases of rapid growth and morphological change of the mouse face. Using the atlases shown in Fig. 3, we registered each subsequent age together by Procrustes superimposition (e.g. E10 to E10.5 and E10.5 to E11) to calculate the facial shape difference. This superimposition method removes size difference between specimens (Fig. 5A,B). We then computed a Pearson correlation between the shape difference and the proliferation in the previous age (Fig. 5C). Shape change was largest between the E10.0 and E11 time points. The overall correlation rate for the younger ages approached 0.75, implying a strong correlation between shape change and morphological change. By E11.0-E11.5, however, this has decreased to around 0.4. The decrease in the older age group is likely due to a combination of decreased shape change between E11 and E11.5 and an overall decrease in the proportion of proliferating cells in the facial region (Fig. 3A).

**Fig. 6.**
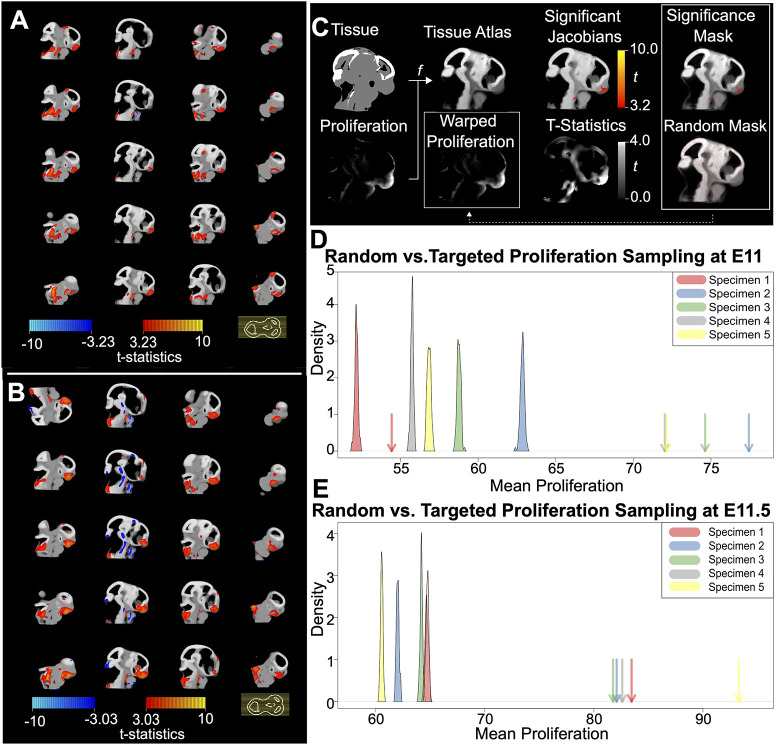
**Voxel-based morphometry for whole embryo heads.** (A,B) Statistical parametric map showing sagittal slices of craniofacial regions that undergo significant shape change from E10.5 to E11 (A) and from E11 to E11.5 (B). The slice legend is displayed on the bottom right, with each yellow line corresponding to an individual slice in the volume. Hot colors (red-yellow scale) indicate voxel expansion, whereas cold colors (blue-teal scale) represent voxel shrinkage. Shape change between time points is displayed on an average shape space. (C-E) Localizing the relationship between cell proliferation and shape change in anatomical context. (C) Overview of the image analysis workflow. We non-linearly registered each tissue volume to a novel tissue atlas, and used the corresponding transformation to warp the proliferation volume into the atlas space. Next, we calculated the Jacobian determinants of the tissue registration field and visualized significant stage-related shape deviations (t-values) with a parametric map. The red-yellow t-value scale indicates levels of increasingly large shape change. Next, we exported a volume of t-statistics to generate a shape significance mask (i.e. voxels with a t-value above the significance threshold), as well as a set of randomly sampled masks (i.e. voxels with randomly sampled t-values). The atlas masks are then overlaid (dashed line) onto each warped proliferation volume to relate proliferation to shape change in the anatomical context. (D,E) Density plots comparing the mean proliferation of *n*=100 randomly sampled masks (bell curves) against the mean proliferation of the significance mask (arrows) for each specimen. E11 and E11.5 are displayed separately.

Next, we sought to directly test the hypothesis that proliferation in regions of significant shape change was larger than what could be expected by chance. This hypothesis is important because if proliferation is a driver of morphological change, it would be predicted that regions of increased rates of shape change should also have increased rates of proliferation. In order to test this, we used additional non-linear voxel-based morphometrics methods to identify regions of local tissue expansion or contraction (representing regional morphological changes) between atlases generated from each age group (Fig. 6A). As expected based on the principal component plot, no regions of expansion or contraction were identified as significant between E10 and E10.5 using these methods. We did not detect any stage-related shape effects at E10, likely due to sample size. However, we observed that E11 and E11.5 contained 627,878 voxels (10.4%, *P*<0.05, t=3.23) and 769,245 voxels (12.7%, *P*<0.05, t=3.08) with significant determinants, respectively. The Dice coefficient for the resulting shape significance masks was 0.57, indicating substantial overlap (Fig. 6A). The largest and most consistent shape changes can be traced to the frontonasal prominences, which undergo immense mediolateral expansion towards the midline during mid-gestation. We then compared proliferation in the expanding regions to an equal number of randomly selected voxels (Fig. 6D,E). Interestingly, we observed that the mean proliferation intensity within these shape significance masks fell outside the distribution of randomly sampled intensities for every specimen. While the mean intensity of the randomly sampled proliferation masks at E11 was 57.26, the mean intensity of the shape significance masks was 70.14. This represents a 22.5% increase in average proliferation at sites of major shape change relative to random sites. Similarly, the mean intensity of the randomly sampled masks at E11.5 was 63.23, whereas the mean intensity of the shape significance masks was 84.64, showing a 33.9% increase in average proliferation. This result shows that there is more proliferation in regions undergoing expansion than would be expected by chance.

**Fig. 7.**
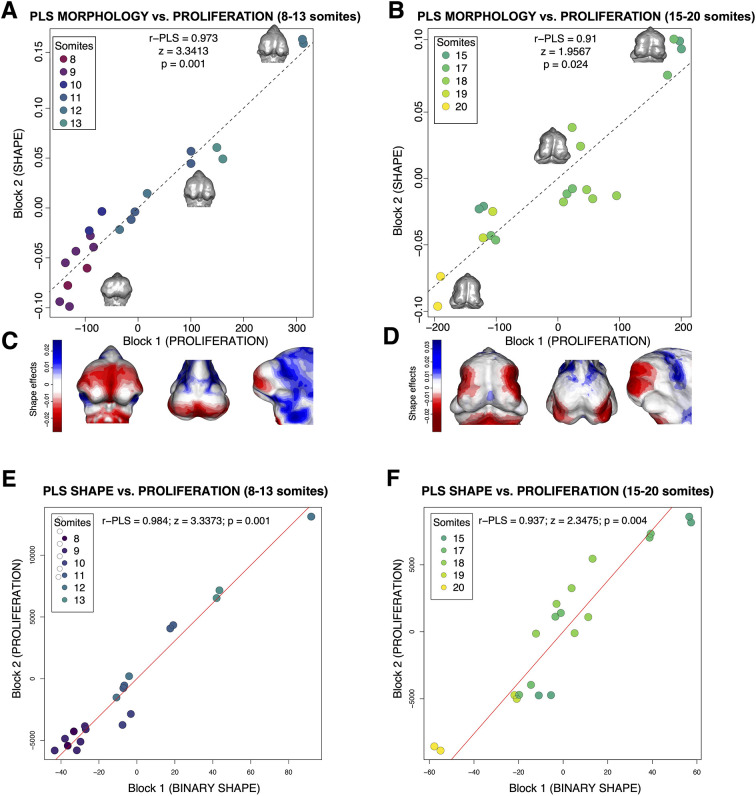
**Quantitative relationship between morphology and spatial patterning of proliferation.** (A,B) Two-block partial least squares (PLS) analysis of Procrustes shape coordinates and proliferation in younger embryos (8-13 somites; A) and in older embryos (15-20 somites; B). Morphs within the scatterplot were constructed from Procrustes coordinates for each specimen. (C,D) Morphs were constructed from Procrustes coordinates for each specimen, and heatmaps depict shape changes associated with changes in proliferation along the first axis (latent variable of shape block), which moves more toward the maxilla with increasing age. (E,F) Two-block PLS analysis using binarized shape data. Note that the binary shape and landmark based shape results are highly similar. *n*=10 per age group set; left and right sides were mirrored and analyzed independently.

To determine whether spatial patterning of proliferation drives ontogenetic variation in morphology, we examined the relationship between the 3D volumetric patterning of proliferation and external morphology among individual embryos across the entire age range of the sample. Our initial two-block partial least squares (PLS) regression for the entire sample showed a very strong relationship between proliferation and morphology (r=0.97, z=2.161, *P*=0.001). To determine whether variation in morphology among individuals correlated with the patterning of proliferation, independently of this strong ontogenetic trajectory, we examined the correlation between the residuals for proliferation and morphology with respect to somite stage. This yielded a much weaker and statistically non-significant correlation (r=0.607, z=1.507, *P*=0.072). However, this analysis is complicated by the clear and strong separation in the patterning of proliferation between the younger and older portions of the ontogenetic range (see Fig. 3B,D). Most of the shape changes associated with proliferation are driven by earlier stages (8-14 somites) and the relationship between proliferation and morphology is different between these two time ranges. Accordingly, we split the sample into the two groups apparent in the PCA plots for both shape and proliferation and then performed PLS regressions within those groups. The resulting two-block PLS analyses revealed strong relationships between the patterning of proliferation and morphology within each age group (Fig. 7). The relationship was somewhat stronger in younger embryos (8-13 somites) (r=0.973, z=3.3.41, *P*=0.001; Fig. 7A). Within this sample, shape changes associated with proliferation were mostly localized to the medial nasal region. In contrast, the PLS in the older embryo group (15-20 somites) showed a slightly weaker correlation between proliferation and shape changes (r=0.91, z=1.957, *P*=0.024; Fig. 7B). Here, shape changes associated with proliferation were mostly associated with maxillary growth.

### Using this method to separate genotypes in a mutant mouse line

**Fig. 8.**
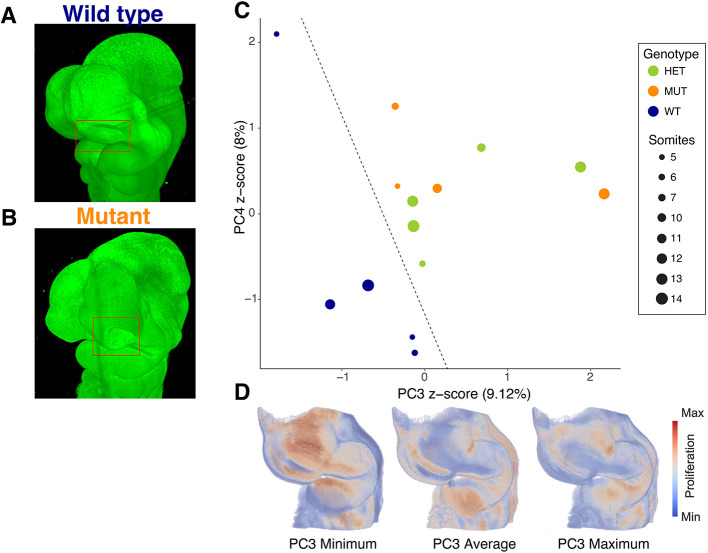
**Separation of genetic mutant lines using SVM.** (A,B) Wild-type (A) and age-matched Unicorn (B) embryos stained for total nuclei (Nuclear Green). Note the subtle difference in shape of the nasal processes in the boxed area. (C) The 14 samples for the Unicorn dataset shown in the PC3 and PC4 from the PCA of the proliferation dataset. These two PCs are the most discriminating PCs according to the coefficients obtained by the linear support vector machine (SVM). The dashed line shows the hyperplane separating the wild-type samples from the other classes (*P*=0.0219 for the permutation test). *n*=4-5 specimens per genotype, 14 total. One side was selected for analysis and no mirroring was used. *P*-value from the SVM model (permutation), *P*=0.02. HET, heterozygotes; MUT, homozygous mutants; WT, wild type. (D) Transformation of the PC3 values to highlight how proliferation is changed across PC3. PC3 maximum: 2.1657; PC3 minimum: −1.7886.

In order to see how this method would perform on mutant mice and to determine the sensitivity of the method, we examined the ethylnitrosourea (ENU)-generated mouse line Unicorn (Lantz et al., 2020) (https://www.informatics.jax.org/allele/MGI:5446162). These mice have mutations in two tightly linked genes, *Raldh2* (*Aldh1a2*) and *Leo1*. The mutants and heterozygotes both have subtle phenotypes and all three genotypes separate on a PCA of eight landmarks around the facial prominences. Overall, from a multi-variate ANOVA, genotype accounts for only 5.5% of facial shape after accounting for age/size-related variation. By shape, the genotypes separated on PC2 and PC3 (Fig. S3). Looking at the proliferation data across the whole head, we find that visually the wild-type embryos separate from the heterozygous and null embryos along PC3 and PC4. When we built a support vector machine (Pedregosa et al., 2011) model to separate the genotypes using PC1-PC5 of the proliferation PCA, we were able to separate the wild-type embryos accurately with a *P*-value of 0.0219 using a permutation test (Fig. 8). Further analysis of the proliferation patterns associated with PC3 showed overall decreased proliferation in the whole nasal capsule and maxilla, with the strongest changes in the lateral nasal process. Although these images do not show an exact proliferation pattern for each embryo, they give an overall idea of the patterns of change.

## DISCUSSION

LSFM offers new opportunities to investigate cell biology within its 3D context, but these opportunities have been difficult to realize due to challenges of quantifying the outcomes. Here, we present a method to overcome some of these challenges and apply it to quantification of the relationship between proliferation and morphology during the events of primary facial morphogenesis in mouse embryos. We place our results in the context of both understanding the role of proliferation in determining facial shape but also in terms of the future tool development needed to push the field forward.

### Structure of growth

Morphogenetic mechanisms drive not only the large changes in morphology over ontogeny but also differences in stage-specific morphology among individuals, including structural birth defects. To study the cellular basis for among-individual variation, it is necessary to quantify morphology and cellular dynamics simultaneously in individual embryos. This contrasts with the common approach of obtaining cellular and phenotypic data in different batches of embryos from the same treatment group or genotype. Without this direct comparison, it is impossible to understand the direct relationship between the cellular process and morphology. The goal of this study was to set out a framework for this type of analysis and to show a real-world example of how this can work. Excellent methods currently exist for high-resolution quantification of morphology in embryos using computed microtomography (Wong et al., 2015; Hallgrimsson et al., 2015) or OPT (Sharpe, 2004; Sharpe, 2003). Existing methods also allow quantification of gene or protein expression along with morphology for the same embryo (Xu et al., 2015; Green et al., 2017, Marchini et al., 2021), but there are few available tools to directly relate morphology to cellular level processes. One reason for this is that quantification of cellular dynamics from a 3D volume in a highly repeatable fashion such that it can be performed on more than a single embryo dataset has proven challenging as this requires anatomical localization of cellular parameters and, preferably, accurate segmentation of individual cells in a 3D anatomical context.

Altering proliferation is, theoretically, one way to alter morphology during development and to generate differences in morphology among individuals at a particular developmental stage (Green, 2022). As organisms grow, the number of cells increases. Spatially homogeneous proliferation would result in spherical growth. More rapid proliferation in some regions of a structure compared to others could generate shape change during morphogenesis. Secreted signaling factors are known to influence spatial patterning of proliferation in a variety of contexts (Duronio and Xiong, 2013). However, spatiotemporal patterning of cell proliferation is not the only potential cellular mechanism for generating morphological variation in growing embryonic tissues (Green, 2022). Planar cell polarity, for example, can produce morphogenetic changes via directional bias in proliferation (Gray et al., 2011). Boehm et al. (2010) showed, using a combination of 3D quantification of proliferation and modeling, that such directional cell behaviors are necessary to explain vertebrate limb morphogenesis. Previously, our work showed that altering Fgf signaling created dysmorphologies that were associated with disrupted polarity of mesenchymal cells in the face (Li et al., 2013). Cell migration is also essential for morphogenesis and has been studied in many developmental contexts. In the neural crest, for example, multiple mechanisms involving secreted factors, extracellular matrix and physical forces interact to produce dramatic changes in cell position and gross morphology (Bronner-Fraser, 1993; Shellard and Mayor, 2019). Even here, though, patterning of cell proliferation within migrating streams of neural crest cells plays a role (Kulesa et al., 2008). Finally, physical forces influence morphogenesis at multiple levels ranging from interaction with extracellular matrix (Daley and Yamada, 2013; Linde-Medina et al., 2016), among cells due to regulation of cell adhesion (Lecuit, 2005; LeGoff and Lecuit, 2016), and physical interactions between tissues such as the neural tube and facial prominences during morphogenesis (Marcucio et al., 2015; Parsons et al., 2011). These alternative and potentially interacting mechanisms for morphogenesis suggest the need for the development of methods to determine objectively the relative contribution of spatiotemporal variation in cell proliferation to fully understand the cellular bases of morphogenesis.

The morphogenesis of the face is complex, likely more so than the limb, because the prominences must grow in different directions and at rates that allow for them to meet and fuse. When this fails to occur, the result is an orofacial cleft and variable disruption of subsequent facial development (Diewert and Wang, 1992). For these reasons, it seems likely that the cellular processes that underlie this growth must be spatiotemporally patterned within the facial prominences. If this applies to cell proliferation, then proliferation should not be homogeneously distributed throughout the volume of growing tissue but rather concentrated in those areas that are growing most rapidly. At each stage of embryonic development, as well as across stages, there should then be a correlation between the 3D spatial pattern of cell proliferation and morphology. This is the hypothesis that we set out to test with the novel methods presented here.

We show that our method is able to separate genotypes in a mutant mouse line previously identified as having subtle differences in proliferation (Lantz et al., 2020). However, this difference is subtle, and genotype only accounts for a small percentage of the embryo-by-embryo differences in proliferation. This is to be expected for proliferation by itself only playing a moderate role in the establishment of morphology. The paper describing the Unicorn mice observed decreased proliferation primarily in the caudal edge of the medial nasal process mesenchyme. They did not examine the lateral edge (Lantz et al., 2020). Although it is difficult to directly compare spot methods to our method, we observe more widespread differences. This was unsurprising as the shape changes are throughout the nasal capsule, so it is logical that there are more proliferation differences than were captured in the original study. Further studies will be necessary to give more detail to these results and understand how each of these potential mechanisms contribute to the development of individual morphology.

Our results show clearly that across developmental time (E10 to E11.5) during facial morphogenesis, there is a strong correlation between the spatial pattern of proliferation and morphology. This pattern is not linear across this range, with distinct patterns of shape change and similarly distinct spatial patterns of cell proliferation in the earlier compared to the later part of this range (Fig. 7). When the relationship between shape and the spatial patterning of proliferation is analyzed separately within each of these two time periods, strong relationships emerge.

Given the finding that spatial variation in proliferation correlates with shape across developmental time, it is surprising that we found that proliferation within the growing prominences during the earlier part of this period was not strongly spatially patterned. We had expected, for example, to find elevated proliferation in regions adjacent to known sources of proliferative signals secreted in regions of ectoderm, such as Fgf8 (Crossley and Martin, 1995; Marcucio et al., 2005) and Shh (Hu and Marcucio, 2009). Instead, we observed a fairly homogeneous distribution of proliferation throughout the volume of the facial prominences that one would expect would result in a pattern of isometric increase in volume rather than change in shape over time. There are two potential explanations for this finding. The first is that within a rapidly growing volume only very small differences in relative rate or proliferation are necessary to produce change in shape. The fact that we observed a correlation between the pattern of proliferation and shape is consistent with this explanation. Alternatively, there may be factors other than proliferation that are influencing facial morphogenesis at this stage, such as those discussed above. These explanations are not exclusive and further work that integrates modeling as well as investigation of molecular markers for other aspects of cellular dynamics of growth are necessary to disentangle them. As part of this study, we also investigated apoptosis, but due to co-staining with vasculature and lack of overall signal in the facial prominences at the earlier time points, we did not include these results in this paper (Fig. S4).

Our finding that proliferation is surprisingly homogeneously distributed is relevant here as this means that loss of proliferation from localized regions may still affect morphological development. The fact that we are able to separate genotypes by proliferation pattern is important as it implies that this method is highly sensitive even though the differences are small. With larger samples, this result suggests the potential to conduct more detailed analyses that connect spatiotemporal patterning of proliferation to ontogenetic and among-individual variation in morphology. The ability to test such hypotheses is still limited by technology. Imaging at 25× magnification and preferably higher is required to evaluate planar cell polarity quantitatively (Alladin et al., 2020; Revenu et al., 2014), for example. Here, the limitation is on the computational side as the resulting images would exceed 1 TB per embryo and registration and processing of such images for whole faces is currently prohibitively resource intensive.

### LSFM as a tool

Although LSFM imaging is becoming more prevalent as a tool for developmental biology, there are many challenges that users of this technique must overcome to apply this method more generally. One issue is that beyond a certain size and tissue density, such as mouse embryos older than E8.5, live LSFM imaging is not feasible as the tissue must be cleared (McDole et al., 2018). Clearing and sample preparation presents significant challenges, although there is an increasing diversity of clearing techniques available. LSFM imaging has even been used to image whole juvenile mice after clearing (Tian et al., 2021). Data processing is also a significant challenge as computational time increases exponentially with resolution or object size at the same resolution. One crucial processing step is the accurate identification of individual cells within an image in order to quantify a cellular marker in relation to the total number of cells. Our team has had recent success retraining a CNN (U-net) to process embryo LSFM images (Lo Vercio et al., 2022). We developed a method that efficiently segments tissues, nuclei and proliferating cells in light-sheet images of whole embryonic heads from E9.5-E10.5 mouse embryos while also dealing with minor LSFM artifacts, such as blurriness or tissue depth variation. Tissue- and nucleus-level analysis becomes more challenging as the size of the tissue sample or embryo imaged increases as image artifacts, such as signal loss due to staining defects and laser penetration, become more pronounced and complex with increasing size. Thus, internal structures, such as the neural tube, are prone to image defects, interfering with its human and computational analysis.

A major issue for the analyses undertaken here is the potential for errors in segmentation. Such errors occur for two reasons. The first is that staining and clearing tends to work better close to the exterior of the sample, which results in depth of tissue-related artifacts. Similarly, whole-mount immunohistochemistry is affected by tissue depth due to variation in antibody penetration (Yokomizo et al., 2012) and this results in loss of signal for interior structures. For this reason, total nuclear counts are included only in the analysis of the facial prominences for the earlier part of the studied age range (E10-E10.5), and total proliferation data was used more extensively in this work. Further, as we approach the resolution limits of individual nucleus detection, apparent merging of nuclei will bias the segmentation process. For this reason, we used a voxel-based approach rather than a single-cell approach for the quantification of the proliferative fraction (Fig. 4). Also, each proliferation map was normalized before computing the proliferation atlases (Fig. 3A) and performing individual-level analyses (Figs 3D and 7). This was to reduce potential effects of inter-sample variability of the proliferation marker. Further work in improving staining, clearing and nucleus and proliferation segmentation is needed to increase the detail and accuracy of the 3D models constructed within the proposed framework.

A final issue relates to the computational resources required to quantify cellular dynamics in a morphological context. Our method involves registration of each volumetric image to a common atlas in order to place both the morphology and the spatial distribution of cells into a common shape space. Atlas generation for early mouse development is a computational challenge due to the high amount of shape variation among embryos (Wong et al., 2015; Devine et al., 2020; Devine et al., 2022). Improvements to atlas generation will require larger sample sizes as well as tighter control of developmental time by increasing the representation per somite stage or by more refined methods of staging. In this work, the more pronounced image artifacts (blurriness, signal loss) in the older embryos (E10.0-E11.5) resulted in poorer performance for the U-nets for tissue segmentation compared to E9.5-E10.5 embryos. This occurred even when U-nets were re-trained with images from our current data, using a larger patch size to compensate for larger embryo size than previous work and to augment tolerance to artifacts. As a result, user intervention was required to correct 3D tissue models in cases where tissues were misclassified. These segmentation errors, in association with the limited sample size and high shape variation during early mouse development limited the degrees of freedom of the registration at the time of building atlases, to restrict the introduction of noise to them (i.e. developmental exceptions in samples, unreal structures or processes due to imaging artifacts, among others). Current work is focused on solving or mitigating these issues so that we can apply non-linear registration methods to the atlas construction, to produce atlases with a higher level of detail while keeping certainty, as well to study phenotypic variations. This will lead to more accurate atlases, better quantification of proliferation maps, and improved ability to handle large and shape-diverse samples of embryos.

### Conclusion and future directions

This work represents a substantive step in the direction of building the toolkit necessary for quantitative integration of analyses across multiple levels of genotype-phenotype maps. Although there are existing methods to quantify gene expression, cellular dynamics, histology and external morphology in isolation, there few methods enable the simultaneous quantification of more than one of these levels in individual embryos. Such efforts are essential in order to create a mechanistic understanding of the generation of phenotypic variation by developmental processes. Although many challenges remain, the method presented here can support the systematic analysis of cell proliferation and morphology in complex morphogenetic contexts such as the development of the vertebrate face. Future efforts will refine this method but also find ways to incorporate additional levels, such as gene expression, spatial transcriptomics and other multiple-molecular marker methods within similar anatomical registration-based frameworks.

## MATERIALS AND METHODS

### Animal generation

Wild-type C57Bl6/J mice from The Jackson Laboratory were used to generate the mice for the normal growth studies. These experiments were approved by the University of Calgary IACUC (B.H.). The Unicorn (b2b1941Clo) mouse line has ENU-induced missense mutations in *Raldh2* and *Leo1*. Mice used in this study had either heterozygous or homozygous mutations in both genes or were wild type at both loci. These mice were also maintained on a C57Bl6/J background. The original Unicorn line has been maintained by outcrossing to wild-type C57/Bl6 animals for more than ten generations (Lantz et al., 2020), and the phenotypes continue to segregate with the ENU-mutagenized alleles of *Raldh2* and *Leo1*. These mice were bred at the University of Pittsburgh and at The University of Saskatchewan and the experiments were approved by their respective IACUC bodies (R.M.G./H.S.-R.).

### Clearing and staining

Dams were euthanized by isoflurane overdose, and embryos were harvested between E9.5 and E11.5. Following harvest, embryos were washed in PBS for 30 min and fixed overnight in 4% paraformaldehyde at 4°C. Embryos were cleared with a modified CUBIC protocol to remove lipids and heme while preserving morphology, based on Susaki et al. (2015). Only typically developing embryos were used for further examination.

Following overnight fixation, samples were washed for 24 h in PBS to remove blood. Samples were serially dehydrated in increasing concentrations of methanol in PBS (25%, 50%, 75%, 100%) for 30 min each. Samples can be stored long-term at this point in 100% methanol at −20°C. Samples were permeabilized in 5% H_2_O_2_ in 100% methanol overnight at 4°C. Samples were rehydrated in decreasing concentrations of methanol in PBS (100%, 75%, 50%, 25%) for 30 min each, followed by a 2 h wash in PBS. Samples were incubated overnight in 1:1 PBS:CUBIC-1 (25% urea, 25% Quadrol, 15% Triton X-100), then in 100% CUBIC-1 until transparent (∼3-5 days), with shaking at 60 rpm at 37°C. Once transparent, samples were rinsed twice in PBS for 2 h, then once more overnight to stop clearing. Samples were then blocked for 36 h in 5% goat serum, 5% DMSO in 0.1% sodium azide in PBS at 37°C to prevent nonspecific binding.

For immunostaining, samples were incubated in anti-Histone H3 (pHH3)-phospho 10 antibody conjugated to Alexa Fluor^®^ 647 (Abcam, AB_330212; 1:200) for proliferation staining and anti-cleaved caspase-3 (Asp175) (D3E9) rabbit monoclonal antibody conjugated to Alexa Fluor^®^ 594 (NEB, 8172S; 1:200) for apoptosis staining. Antibodies were diluted in 5% DMSO in 0.1% sodium azide in PBS together with Nuclear Green (1:4000) for nuclear staining and incubated for 5 days at 37°C. Following staining, samples were washed several times in 0.5% Tween 20 in PBS to remove excess antibody. Samples were beheaded, and heads were embedded in 1.5% low melting point agarose and incubated in CUBIC-2 (25% urea, 50% sucrose, 10% triethanolamine) for 24 h. Tails were also embedded in order to stage embryos after imaging.

### Imaging

Light-sheet images of agarose-embedded samples were obtained with a Zeiss Lightsheet Z.1 microscope. Heads were imaged at 5 µm intervals with 0.9 µm resolution with a 5× objective in CUBIC-2. Tails were separately imaged at 7 µm intervals with 2.5 µm resolution for manual counting of tail somites as a measure of embryonic age (Chan et al., 2004). Image stacks were taken with a 10% overlap and stitched together in ZEN Blue (Zeiss).

### Automatic analysis workflow

#### Segmentation

A U-net for tissue segmentation (Fig. S1) was trained using Nuclear Green images (4096×4096 downsized to 256×256), 21 images from E11.0 embryos and ten images from E11.5 embryos, annotated by two expert observers. The U-net was trained using an equally weighted combined loss function of soft-Dice and categorical cross-entropy, RMSprop optimizer with learning rate 0.001, for 100 epochs. Training data augmentation based on deformations (flipping, affine deformations) and based on intensities (blurring, brightness, contrast) was performed on the fly (https://github.com/aleju/imgaug; accessed 10 July 2020). For a test set of eight images from a E11.0 embryo and ten from a E11.5 embryo, annotated by a third observer, the U-net reached an accuracy of 0.806 and a Dice score of 0.727. For the segmentation of the proliferation marker (pHH3) and nuclear channel (Nuclear Green), U-nets (Falk et al., 2019) were used, particularly those with retrained architectures in a previous work of our group (Lo Vercio et al., 2022), for these cell segmentation tasks.

#### Registration for atlases

To build general shape and proliferation maps at each developmental age (E10.0, E10.5, E11.0 and E11.5), one sample per age was selected as reference. Then, a landmark-based rigid registration was performed between each of the remaining samples in the age group and their reference sample, using five landmarks placed in the head (Fig. S2A) by an expert observer (Devine et al., 2022) using SimpleITK in Python (Lowekamp et al., 2013). Then, the resulting transformation was applied to the corresponding tissue segmentation. A groupwise-affine registration was performed among the ten registered tissue segmentations using SimpleElastix in Python. It was computed using multiresolution registration with number of resolutions 5, maximum iterations 30,000, linear interpolator, and nearest neighbor interpolator for resampling (Fig. S2B) (Marstal et al., 2016; Devine et al., 2020). Finally, the voxels of the shape atlas were labeled as background, mesenchyme or neural ectoderm using majority voting, implemented in MATLAB (Fig. 3A).

The chain of rigid and affine registrations of each sample was later applied to the corresponding volumes of nuclei and proliferating cells (Fig. S2C,D). The mean proliferation per voxel was computed using a 0.15 mm×0.15 mm×0.15 mm window. This proliferation map of the sample was normalized using percentile-based equalization (Weigert et al., 2018) (Fig. S2E). The mean proliferation map for each age was obtained by averaging the proliferation maps of the five embryos and their mirrored maps (Fig. 3A).

#### Registration for bulk analysis

To analyze the shape and proliferation variation among all the samples used in this work, 37 landmarks were placed in the face of the 20 samples and their mirrored volumes by an expert observer (Fig. S2B) (Percival et al., 2014). Then, a landmark-based similarity registration (translation, rotation and scaling) was performed using VTK in Python (Schroeder et al., 2006) between each sample and one E10.5 sample used as the reference (transformed as in the previous section). These transformations were later applied to the corresponding proliferation maps.

#### Voxel-based study

Using the tissue and proliferation maps produced by the registration process described above, we proceeded to extract the shape and proliferation in the face of the registered embryos. To build this mask, the 40 mesenchyme segmentations were extracted and voxels labeled as mesenchyme more than 30% of the time were assigned to a primary mask. Then, voxels further away than 0.39 mm from the surface of this mask were excluded. Finally, an expert observer manually refined the mask using 3D Slicer leaving only voxels belonging to the face. With this mask, the proliferation map values were extracted for the 40 samples.

#### Landmark-based study

We analyzed and visualized facial shape using geometric morphometrics methods. We performed a generalized procrustes superimposition analysis (Gower, 1975) to extract the aligned Procrustes shape coordinates from the manual landmark dataset (to focus on shape differences only on the face and not the whole head; Fig. 3C), using the package geomorph (version 4.0.4; https://cran.r-project.org/package=geomorph; Baken et al., 2021) in R (https://www.R-project.org/). The Procrustes shape coordinates represent each specimen's shape, and we ordinated these with a PCA to visualize the axes of maximum shape variation and their association throughout ontogeny (using tail somite number as a proxy for age), with the R package geomorph (Fig. 3B). We also performed a PCA on the flattened matrix from the proliferation array in order to be able to visualize differences in proliferation associated with age (Fig. 3D).

To assess the degree of association between face shape and proliferation patterns, we performed a two-block PLS (Rohlf and Corti, 2000) analysis using the R packages Morpho (Schlager, 2017) and geomorph. PLS latent variables were calculated as the linear combinations of the Procrustes shape coordinates (Block 2) and the flattened proliferation matrix (Block 1), which maximized the covariance between the two blocks. We plotted an ordination PLS scatterplot, colored by the number of tail somites (indicating age), displaying the first latent variable for both the shape dataset (Block 2) and the proliferation dataset (Block 1), using geomorph (Fig. 7). We split the embryonic dataset into two age-relevant groups, based on their distinct grouping in both the facial shape morphospace (Fig. 3C) and proliferation patterns (Fig. 3D). Meshes to produce morphs were obtained from smoothed atlases using morphological closing. Finally, we generated morphs using the shape changes associated with proliferation changes, using the R package Morpho (Fig. 7C,D). We repeated the PLS analysis by replacing the Procrustes shape coordinates with the flattened binarization of the tissue segmentation matrix (Fig. 7E,F).

#### Single tissue analysis

Based on our whole-head proliferation atlas, the frontonasal and maxillary prominences were identified as the most actively proliferating mesenchymal tissues in the face between E10.0 and E11.0, and because of that were selected for tissue-specific analysis. Tissues were segmented on the atlas in 3D Slicer and back-propagated to each sample in the group. Total and proliferating nucleus volumes were calculated by segmenting cells as described above using a connected components method. Nuclear volumes were used instead of counts to reduce segmentation errors in dense tissues, especially in the Nuclear Green stain. Neighboring cells in dense stains tended to be under-segmented; by summing total volumes of segmented nuclear voxels, there was no need to identify which cell they belong to, removing a potential source of error. Cells below the 1st and above the 99th percentile in volume were considered segmentation artifacts and excluded from analysis. Cells were binned by relative position along each tissue axis, and proliferative fraction was calculated as the proportion of cell voxels that also contained a proliferation voxel in each bin. To identify nonuniform trends in proliferation, a Kolmogorov–Smirnov test of uniformity was conducted on each axis.

### Analysis workflow for mutants

#### Segmentation

The workflow described to analyze wild-type data was adapted to analyze mutant data. Tissue segmentation was performed using the proposed U-net in this work (Fig. 1). Segmentation of pHH3 was carried out using Cellpose (Stringer et al., 2021), which was retrained using the patches from Lo Vercio et al. (2022) with the segmentation from its re-trained U-net (Falk et al., 2019) as the ground truth, for consistency with the performance obtained for the wild-type dataset. Then, the mean proliferation per voxel was computed using a 0.15 mm×0.15 mm×0.15 mm window.

#### Registration for bulk analysis

The landmark set of 37 landmarks (Fig. S2B) were placed in the face of the 14 samples. These samples and their landmarks were automatically mirrored. Then, the landmark-based similarity registration was performed between each sample and the same E10.5 sample used as the reference for the wild-type dataset. These transformations were later applied to the corresponding proliferation maps of each sample and its mirrored version.

#### Voxel-based study

Given the heterogeneity of phenotypes in this dataset, along with the wide age range of it, a new mask to retrieve the proliferation in the frontonasal and maxillary prominences was needed. This mask of the mesenchyme tissue was obtained by majority voting of the registered tissue segmentations. Then, to remove imperfections and discontinuities, a binary image closing was performed with a structure element of 40×40×40 pixels. Voxels further away than 0.39 mm from the surface of this mask were excluded. Finally, an expert observer manually refined the mask using 3D Slicer leaving only voxels belonging to the frontonasal and maxillary prominences.

Because of the high asymmetry in the proliferation signal in the original scans, only the half corresponding to the clearest signal side was used. Selecting as a reference the right side of this new mask, if the best signal side of the sample was the left, the right side of the mirrored sample was incorporated in the analysis.

To study differences in the proliferation between the mutant, wild-type and heterozygous samples, a PCA analysis was performed in the set of proliferation volumes (Fig. S3). To determine whether there was a significant difference among their proliferation maps, support vector machines (SVM) were used to classify proliferation in wild-type compared with mutant and heterozygous samples. First, the scores for the first five principal components, which explain 73.29% of the variance were standardized (z-score). Then, the linear SVM for classification provided by scikit-learn (Pedregosa et al., 2011) was utilized, using L1 loss, 10,000 iterations and regularization parameter C equal 1, to solve the primal optimization problem. The SVM reached an accuracy of 100% and a F1-score of 1 in this classification task (Fig. 8). To study the significance of this classification task, the permutation test score function provided by scikit-learn was used, with 1000 permutations and the F1-score as the metric to evaluate due to the class imbalance of the dataset, giving a *P*-value of 0.0219.

## Supplementary Material



10.1242/develop.204511_sup1 Supplementary information
