# Supplementary material for: Quantifying the relationship between cell proliferation and morphology during development of the face
Source: Development. 2025 Apr 7;152(7):dev204511. doi: 10.1242/dev.204511 (PMC12045601; doi:10.1242/dev.204511)
Supplement: Supplementary information [file develop-152-204511-s1.pdf]

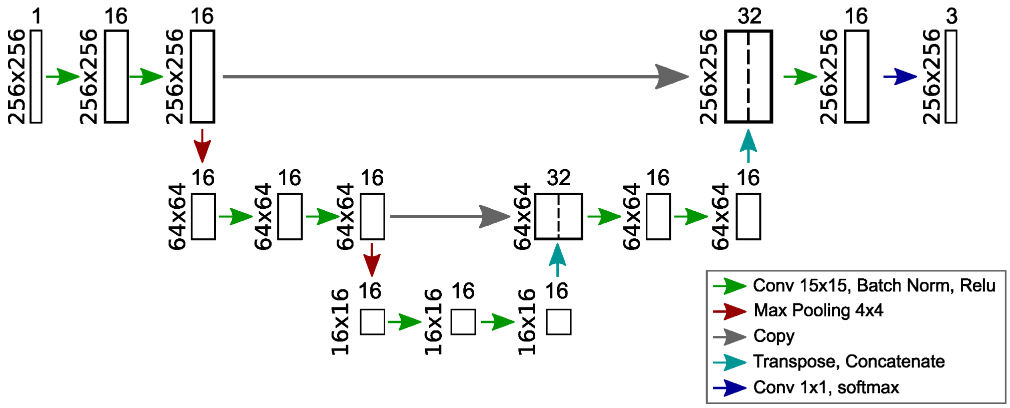

Fig. S1. U-net architecture for tissue segmentation. Implemented in TensorFlow-Keras. It was trained using an equally weighted combined loss function of soft-Dice and categorical cross-entropy, RMSprop optimizer with learning rate 0.001, for 100 epochs. Training data augmenta-tion based on deformations (flipping, affine deformations) and based on intensities (blurring, brightness, contrast) was performed on the fly (A. B. Jung. 2020. Imgaug).

Development • Supplementary information

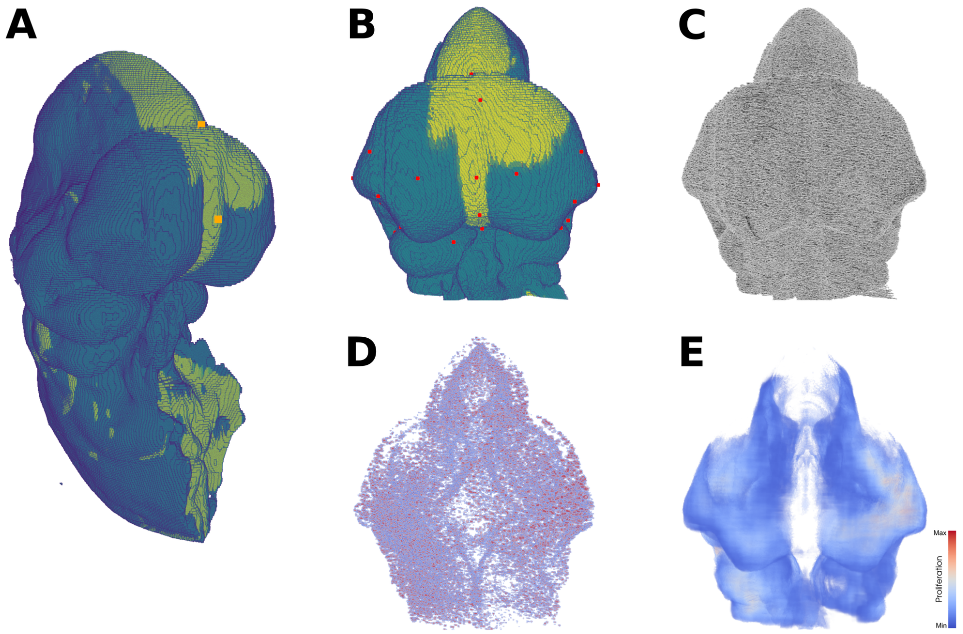

Fig. S2. Example of segmentations, transformations and landmark configurations for a E10.0 sample. (A) Tissue segmentation of the full embryo (mesenchyme in teal, neural ectoderm in yellow) and its five landmarks (orange) for the initial rigid registration to a common reference for atlas construction. (B) Segmentation-based groupwise-affine registration result, for atlas construction. The thirty-seven landmarks used for later bulk analysis are shown in red. (C) Nuclei segmentation isotropic volume after applying the result of the groupwise-affine registration. (D) Proliferating cell segmentation isotropic volume after applying the result of the groupwise-affine registration. (E) Proliferation map in mesenchyme after convolving the volume shown in D and masking with the volume shown in B.

Development • Supplementary information

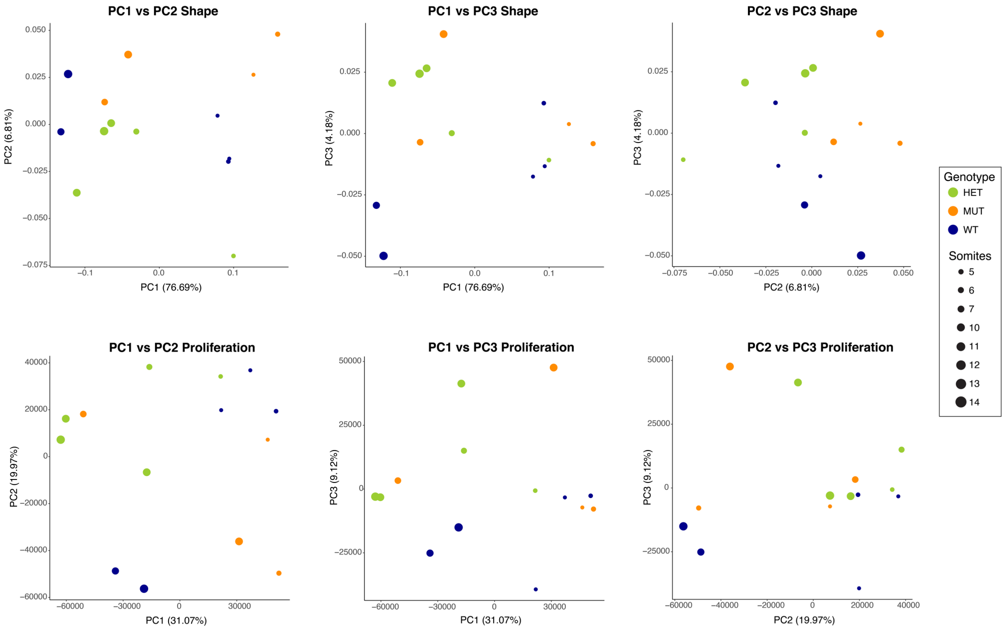

Fig. S3. Principal Component Analysis of the *Unicorn* animals. Animals that are heterozygous for the mutation at both the *RA* and *Leo* loci are shown in green, homozygous for the mutation at both loci are orange and wildtype at both loci are blue. Embryo age is inidcated by dot size. PC1 corresponds tightly with age for both shape and proliferation. PC2 and 3 separate genotypes in the shape data, however it is PC4 and 5 which segregate genotypes in the proliferation data (Figure 7 - main paper).

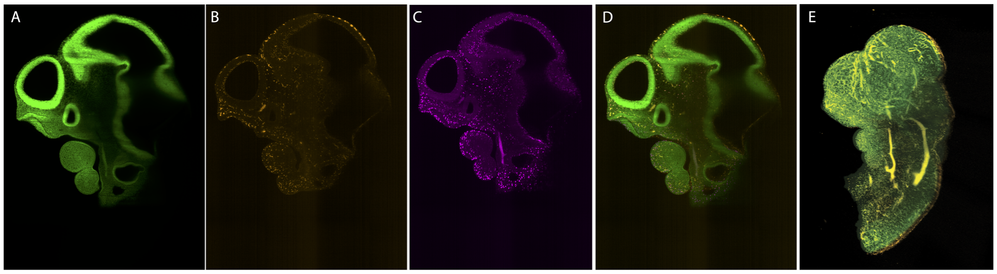

Fig. S4. Section Views for Proliferation and Apoptosis. Lightsheet images of a 9 tail somite C57Bl6/J mouse embryo. A) Total nuclei stained with Nuclear Green. B) Apoptotic nuclei stained with Cleaved-Caspase 3. C) Proliferating nuclei stained with Phospho-Histone H3. D) All Channels. E) Maximum image projection of the whole head

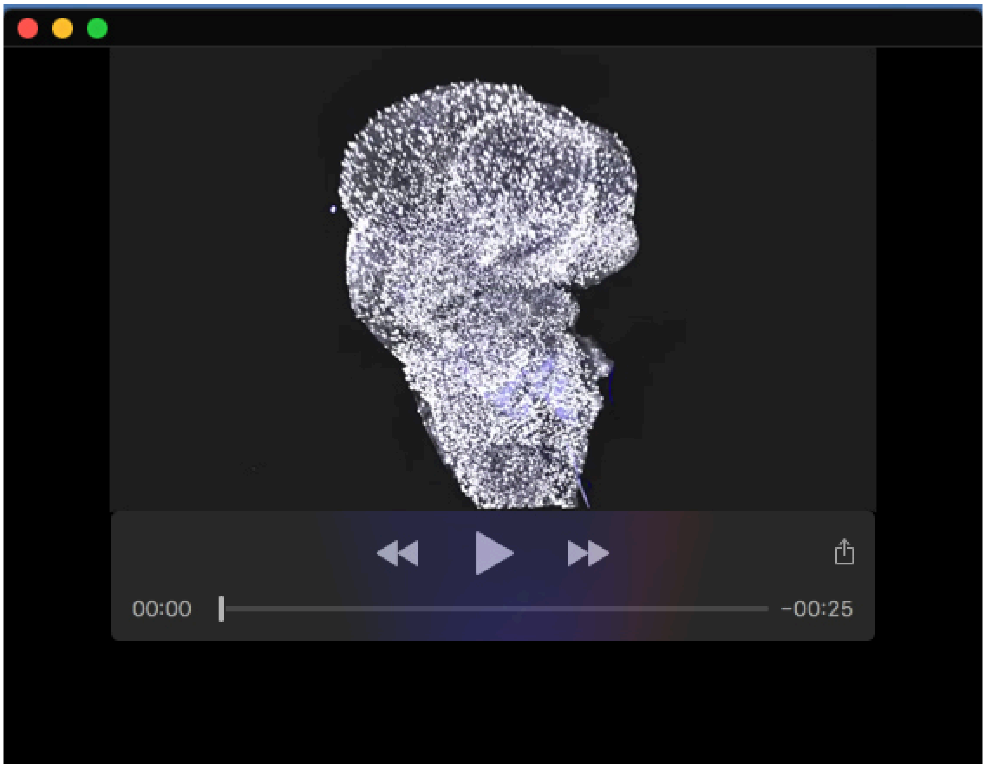

Movie 1. E10.5 embryo stained for Phospho-Histone H3 (white) to stain proliferating cells and Cleaved caspase 3 (blue) to stain apopotic cells.

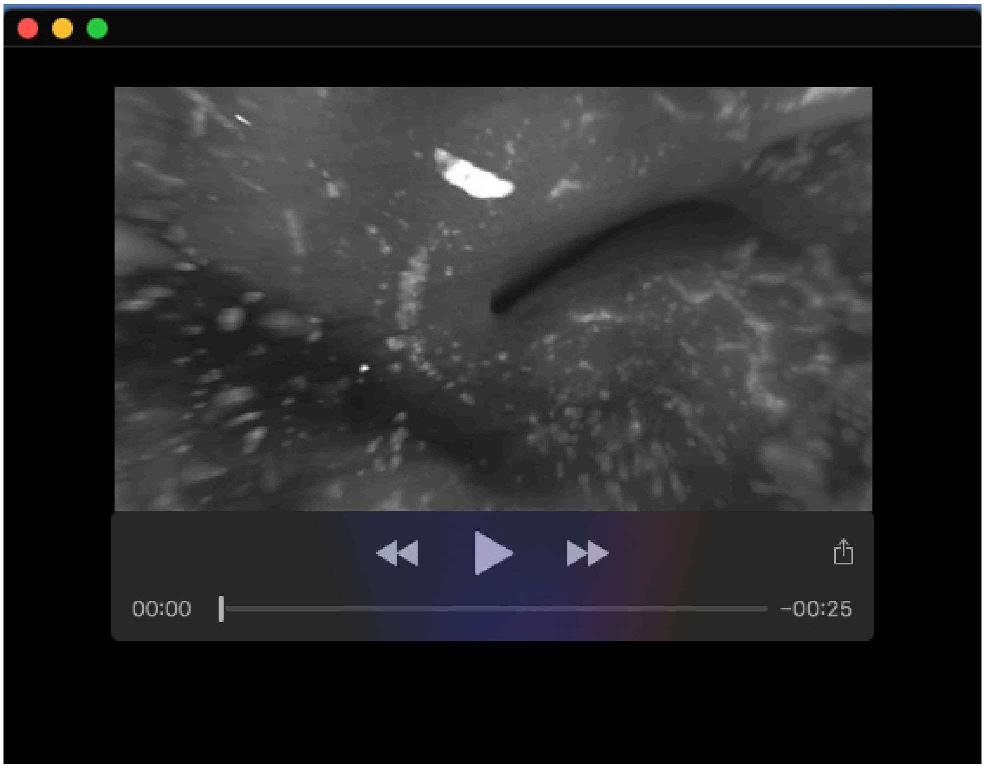

Movie 2. E10.5 mouse embryo stained for all nuclei (Sytox Green; white) and Phospho-Histone-H3 (proliferation; blue).

Development • Supplementary information
