# Supplementary material for: Quantifying the relationship between cell proliferation and morphology during development of the face
Source: Development. 2025 Apr 7;152(7):dev204511. doi: 10.1242/dev.204511 (PMC12045601; doi:10.1242/dev.204511)
Supplement: Supplementary file 1 [file develop-152-204511_review_history.pdf]

Quantifying the relationship between cell proliferation and morphology during development of the face

Lucas D. Lo Vercio, Rebecca M. Green, Andreas Dauter, Elizabeth C. Barretto, Marta Vidal-García, Jay Devine, Marta Marchini, Samuel Robertson, Xiang Zhao, Anandita Mahika, M. Bilal Shakir, Sienna Guo, Julia C. Boughner, Heather Szabo-Rogers, Wendy Dean, Arthur D. Lander, Ralph S. Marcucio, Nils D. Forkert and Benedikt Hallgrímsson
DOI: 10.1242/dev.204511

Editor: Anna-Katerina Hadjantonakis

Review timeline

Original submission:	12 May 2023
Editorial decision:	3 July 2023
Resubmission:	19 November 2024
Editorial decision:	16 December 2024
First revision:	22 January 2025
Accept:	12 February 2025

Original submission

First decision letter

MS ID#: DEVELOP/2023/201981

MS TITLE: Quantifying the relationship between cell proliferation and morphology during development of the face

AUTHORS: Rebecca M Green, Lucas Lo Vercio, Andreas Dauter, Elizabeth C Barretto, Jay Devine, Marta Vidal Garcia, Marta Marchini, Samuel Robertson, Xiang zhao, Anandita Mahika, M Bilal Shakir, Sienna Guo, Julia C Boughner, Wendy Dean, Arthur D. Lander, Ralph S. Marcucio, Nils D Forkert, and Benedikt Hallgrimsson

Dear Dr. Hallgrimsson,

I have now received all the referees' reports on the above manuscript, and have reached a decision. The referees' comments are appended below, or you can access them online: please go to BenchPress and click on the 'Manuscripts with Decisions' queue in the Author Area.

As you will see, the referees express considerable interest in your work, but have some significant criticisms and recommend a substantial revision of your manuscript before we can consider publication. If you are able to revise the manuscript along the lines suggested, which may involve further experiments, I will be happy receive a revised version of the manuscript. Your revised paper will be re-reviewed by one or more of the original referees, and acceptance of your manuscript will depend on your addressing satisfactorily the reviewers' major concerns. Please also note that Development will normally permit only one round of major revision.

© 2025. Published by The Company of Biologists under the terms of the Creative Commons Attribution License (https://creativecommons.org/licenses/by/4.0/).

If it would be helpful, you are welcome to contact us to discuss your revision in greater detail. Please send us a point-by-point response indicating your plans for addressing the referee's comments, and we will look over this and provide further guidance.

Please attend to all of the reviewers' comments and ensure that you clearly highlight all changes made in the revised manuscript. Please avoid using 'Tracked changes' in Word files as these are lost in PDF conversion. I should be grateful if you would also provide a point-by-point response detailing how you have dealt with the points raised by the reviewers in the 'Response to Reviewers' box. If you do not agree with any of their criticisms or suggestions please explain clearly why this is so.

Reviewer 1 Advance Summary and Potential Significance to Field:
This manuscript describes a significant extension of prior research with a relatively sophisticated image processing analysis pipeline based on light sheet microscopy to explore the relationship between proliferation and 3D morphology in mouse embryonic craniofacial tissues. The morphometric approach applied to quantifying spatiotemporal differences in cell proliferation compared to embryonic surface morphology is a valuable advance. Although the work unfortunately but necessarily used fixed tissues, it provides a rigorous approach to beginning to determine the relationship between cell proliferation and morphogenesis and anatomical variation. One surprising finding is that there is a relatively diffuse distribution of proliferation in regions undergoing shape change rather than the expected local foci of proliferation. This approach provides a useful addition to the methodologies needed to quantify contributors to morphogenetic change.

Reviewer 1 Comments for the Author:
This manuscript is carefully documented and presented. The data presented are convincing, and the methodologies are useful.
1. It is unfortunate that the samples had to be chemically fixed such that the contributions of cell migration and PCP could not be evaluated, since the finding of barely more than a 20% increase in proliferation at sites of major shape change compared to random sites seems surprisingly low. That is, even though there was high correlation between regions of proliferation and shape change, the rather low, diffuse pattern of proliferation seems surprising. In that regard, can the authors compare the rate of local tissue volume change to the change in proliferation? That is, can the changing surface morphology be translated to volume change to permit this additional type of quantitative analysis?
2. A valuable adjunct to this work would have been an evaluation of some murine genetic disorder in facial morphology to provide a more direct test of the role of proliferation in shape change, but one can argue that such additional work is beyond the scope of the present presentation of an excellent analysis pipeline.
3. The manuscript preparation seemed excellent, and this reviewer had no minor concerns.

Overall, this solid work warrants eventual publication.
*****Reviewer 2 Advance Summary and Potential Significance to Field:

In their manuscript "Quantifying the relationships between cell proliferation and morphology during development of the face" Green and colleagues use light sheet microscopy and neural network analyses to characterize facial growth in mouse embryos. The authors state that they focused primarily on mesenchymal tissue due to difficulties in segmenting the neural ectoderm. Following segmentation they applied landmark-based morphometric analyses on the faces. PCA found that principal component 1 explained the majority of variance in the facial shape data and the plurality of variance in proliferation. In both instances PC1 separated the E 10 and E 10.5 embryos from the E 11 and E11.5 embryos. They then asked if rates of proliferation across the various axes of the facial prominences would precede morphological changes. They found that, with the exception of the dorsal region of the frontonasal prominence, no significant differences in proliferation density. They then applied a voxel-based approach registering subsequent ages together for shape change and correlating proliferation from the younger age to the transition. Here, they found very strong correlations between proliferation and shape change. They demonstrate that regions of tissue expansion or contraction have greater levels of proliferation then an equal number of randomly

© 2025. Published by The Company of Biologists under the terms of the Creative Commons Attribution License (https://creativecommons.org/licenses/by/4.0/).

selected voxels (a well appreciated control). They end with partial least squares regression for embryos between 8-13 and 15-20 somites to examine the relationship between proliferation and shape. Each group was statistically significant, although the younger group was more so. The work advances the fields of light sheet microscopy, machine learning and morphometrics.

Reviewer 2 Comments for the Author:
The analyses are rigorous and, for the most part, well explained. There are some issues that should be clarified, however:
1). The authors state that they ran into difficulties segmenting neural ectoderm. This is quite vague. They do discuss challenges of light sheet microscopy in the discussion but future research aimed at this tissue would benefit from a more detailed description of the precise difficulties they ran into.
2) In the PCA, PC2 appears to provide some granularity, separating younger embryos out by somite count.
Can the authors elaborate/comment on this?
3) The authors should elaborate further on Figure 5B. I would interpret it to mean that at the transitions being studied ~ 25%, ~45% and ~15% of voxels show growth either outward or inward, but this is not clearly stated anywhere. The authors should state precisely what the data show. Also, is there a reason to combine outward and inward remodeling? Are they equally impacted by proliferation?
4) The statement "…our findings do not support the view that spatial patterning of proliferation accounts for differences in morphology among individuals at a given stage" is misleading. This study is vastly underpowered to detect such small differences, so the findings neither support nor refute such a statement. The authors should remove this paragraph or, if they wish do discuss individual variation, they should simply state that much larger studies are necessary to determine the association of proliferation and individual differences.

Minor issues:

Line 921: Should this read: the relationship "between" the 3D Volumetric patterning…

Line 923: "two block two-block…" should this just be two-block?

*****Reviewer 3 Advance Summary and Potential Significance to Field:
This methods paper tries to tackle one of the holy grails of developmental biology i.e., relating the morphological changes observed in development to the cellular processes that drive/generate them. It uses the emerging lightsheet microscopy (LSM) to obtain cellular level resolution on whole embryo level. At analytical level it leverages another emerging analytical methodology, deep-learning to effective segment cells and tissues. It has the potential to be impactful in the field of morphogenesis and early development, and when executed right allow to investigate cellular mechanisms that result in developmental defects and dysmorphologies in a direct manner.

Reviewer 3 Comments for the Author:

I have couple significant issues with the manuscript.
1. For methods and technique paper, I do not think the paper stands by itself. THere is extensive reference to other published articles (e.g., Unet model for cellular segmentation). I would have preferred to see more technical implementation detail without having to read the previous manuscripts. I would encourage the authors to shorten the really extensive introduction section, which goes on quite a bit of shortcomings of prior imaging modalities (such as microCT and OPT), and actually expand the methods section.
2. I was a little surprised and not entirely clear why the stage atlases were created using only affine registration? Affine transformation is a linear transformation that only accounts for global size differences. Given the rapid pace of development at E9 and E10, I would have expected there are many morphological differences in the embryos (even if they were from timed matings) that will not be sufficiently captured by an affine only atlas building process. I was not able to locate

© 2025. Published by The Company of Biologists under the terms of the Creative Commons Attribution License (https://creativecommons.org/licenses/by/4.0/).

the atlas files to check this for myself. I would strongly encourage the authors to submit their atlases as part of their data repository (and if they did I think it should have been more clear in the manuscript).
3. On Figure 5 the colorbar does not indicate the magnitude of scale. I imagine red are higher magnitude proliferation and blue is lower, but please do not make us guess. Also what is the unit? In general I find the yellow/green color rendering very difficult to follow. Also this being 3D data (and more importantly analysis), I can't interpret what's going on inside the embryo. I think if the authors can provide the actual 3D models (or better yet voxel maps) in an open-source format (since they are already using 3D Slicer for part of their workflow) it will be better than a static 2D image.
4. In some ways it is not surprising to have high correlation between higher proliferation and morphological changes, but it is of course it is still a nice validation. What was surprising to me is the lack of correlation between apoptosis and morphological change. Because it is essentially the flip side of the same argument made by the authors in L1064. It wasn't clear this was not investigated or it was investigated and found not to be significant and hence not reported.

Resubmission

Author response to reviewers

Dear Reviewers,
We want to thank you for your time and patience with this resubmission. In response to your comments, we have added a major portion of additional data where we have repeated the methods using the *Unicorn* mutant mouse line which has a subtle change in the growth of the facial prominences. We have also made revisions across the manuscript, most of which are discussed here.
Thank you again for your time.

Reviewer 1 Advance Summary and Potential Significance to Field:
This manuscript describes a significant extension of prior research with a relatively sophisticated image processing analysis pipeline based on light sheet microscopy to explore the relationship between proliferation and 3D morphology in mouse embryonic craniofacial tissues. The morphometric approach applied to quantifying spatiotemporal differences in cell proliferation compared to embryonic surface morphology is a valuable advance. Although the work unfortunately but necessarily used fixed tissues, it provides a rigorous approach to beginning to determine the relationship between cell proliferation and morphogenesis and anatomical variation. One surprising finding is that there is a relatively diffuse distribution of proliferation in regions undergoing shape change rather than the expected local foci of proliferation. This approach provides a useful addition to the methodologies needed to quantify contributors to morphogenetic change.

Reviewer 1 Comments for the Author:
This manuscript is carefully documented and presented. The data presented are convincing, and the methodologies are useful.

Thank you, we are excited that you found it interesting and hope that we have addressed your concerns.

1. It is unfortunate that the samples had to be chemically fixed such that the contributions of cell migration and PCP could not be evaluated, since the finding of barely more than a 20% increase in proliferation at sites of major shape change compared to random sites seems surprisingly low. That is, even though there was high correlation between regions of proliferation and shape change, the rather low, diffuse pattern of proliferation seems surprising.

© 2025. Published by The Company of Biologists under the terms of the Creative Commons Attribution License (https://creativecommons.org/licenses/by/4.0/).

Response to concern: We agree with this review. Lightsheet microscopy is currently limited by laser penetration into opaque tissues. We are currently exploring alternate ways to understand how other tissue properties are involved in spatial patterning and growth of tissue. The low, diffuse pattern was also surprising to us, and we are also currently exploring other hypotheses that may be affecting tissue out growth in ways that are not observed in this paper, such as directional division.

In that regard, can the authors compare the rate of local tissue volume change to the change in proliferation? That is, can the changing surface morphology be translated to volume change to permit this additional type of quantitative analysis?

Response to concern: Yes, this is a good idea. Thank you for the insight.
Action taken: The analysis that the reviewer suggested is feasible using the materials and methods already included in this work. Voxel based analysis (local tissue volume change analysis) has already been used in Fig. 5 and Fig. 6 in two different ways to relate proliferation in a certain age to the volume change. However, these results do not directly correlate the change in local tissues column to the change in proliferation. To compare/link their rates of change, we plan to use the strategy used in Fig. 7, where we used PLS to relate proliferation and shape change using geometric morphometrics (GMM). Now, instead of GMM in Block 1, we will use the volumetric change (Fig. 6) in this block. We have added this result to Fig. 7, though there is little difference from the morphometric shape.

2. A valuable adjunct to this work would have been an evaluation of some murine genetic disorder in facial morphology to provide a more direct test of the role of proliferation in shape change, but one can argue that such additional work is beyond the scope of the present presentation of an excellent analysis pipeline.

Response to concern: While this has been a massive undertaking, we have added an analysis of mutants to the paper. We have used the *Unicorn* ENU mutant, which has a subtle midfacial phenotype. These results are now figures 8. We hope that the reviewers find this additional data meaningful.

3. The manuscript preparation seemed excellent, and this reviewer had no minor Concerns.

We thank the reviewer for this thorough evaluation of the paper and helping us to improve it.

Overall, this solid work warrants eventual publication.

***** Reviewer 2 Advance Summary and Potential Significance to Field:
In their manuscript "Quantifying the relationships between cell proliferation and morphology during development of the face" Green and colleagues use light sheet microscopy and neural network analyzes to characterize facial growth in mouse embryos. The authors state that they focused primarily on mesenchymal tissue due to difficulties in segmenting the neural ectoderm. Following segmentation they applied landmark-based morphometric analyses on the faces. PCA found that principal component 1 explained the majority of variance in the facial shape data and the plurality of variance in proliferation. In both instances PC1 separated the E 10 and E 10.5 embryos from the E 11 and E11.5 embryos. They then asked if rates of proliferation across the various axes of the facial prominences would precede morphological changes. They found that, with the exception of the dorsal region of the frontonasal prominence, no significant differences in proliferation density. They then applied a voxel-based approach registering subsequent ages together for shape change and correlating proliferation from the younger age to the transition. Here, they found very strong correlations between proliferation and shape change.

© 2025. Published by The Company of Biologists under the terms of the Creative Commons Attribution License (https://creativecommons.org/licenses/by/4.0/).

They demonstrate that regions of tissue expansion or contraction have greater levels of proliferation then an equal number of randomly selected voxels (a well appreciated control). They end with partial least squares regression for embryos between 8-13 and 15-20 somites to examine the relationship between proliferation and shape. Each group was statistically significant, although the younger group was more so. The work advances the fields of light sheet microscopy, machine learning and morphometrics.

Reviewer 2 Comments for the Author:
The analyses are rigorous and, for the most part, well explained. There are some issues that should be clarified, however:

1). The authors state that they ran into difficulties segmenting neural ectoderm. This is quite vague. They do discuss challenges of light sheet microscopy in the discussion but future research aimed at this tissue would benefit from a more detailed description of the precise difficulties they ran into.

Response to concern: We thank the reviewer pointing out this comment and recognizing the potential of a more detailed explanation has to encourage future research.

Action taken:
In the Results section, page 4, line 363 we changed the sentences: "Our analysis focused on the non-neural tissues, primarily mesenchyme, as most of the key events happening during facial morphogenesis happen in mesenchymal tissues. As we ran into difficulties segmenting cells in the highly dense neural tissue, we did not pursue further analysis in this tissue."
To:
"Our analysis focused on the non-neural tissues, primarily mesenchyme, as most of the key events happening during facial morphogenesis happen in mesenchymal tissues. Furthermore, the dense neural tissue along with image artifacts, such as blurriness and signal loss, hinder the proper segmentation of the tissue itself and its cells."

In the Discussion section, page 14, line 1465, we expanded the discussion of the segmentation of deep tissues and punctual staining:
"Tissue- and nucleus-level analysis becomes more challenging as the size of the tissue sample or embryo imaged increases as image artifacts, such as signal loss due to staining defects and laser penetration, become more pronounced and complex with increasing size. Thus, internal structures, such as the neural tube, are prone to image defects, interfering with its human and computational analysis."

2) In the PCA, PC2 appears to provide some granularity, separating younger embryos out by somite count.
Can the authors elaborate/comment on this?

Yes, we have elaborated further by showing how changes along PC1 and PC2 related to the facial shape difference and added these thumbnail figures to the graph axes. We have added additional sentences to the text highlighting the shape differences between the groups. It can be found in Figure 3.

3) The authors should elaborate further on Figure 5B. I would interpret it to mean that at the transitions being studied ~ 25%, ~45% and ~15% of voxels show growth either outward or inward, but this is not clearly stated anywhere. The authors should state precisely what the data show. Also, is there a reason to combine outward and inward remodeling? Are they equally impacted by proliferation

Response to concern: We have rewritten the caption of the Figure 5 and the associated text to clarify how we built the transitions for this analysis and the interpretation of outward and inward remodeling in the context of Procrustes analysis.

© 2025. Published by The Company of Biologists under the terms of the Creative Commons Attribution License (https://creativecommons.org/licenses/by/4.0/).

Figure 5 new text - we have removed the size discussion.

Voxel-based analysis of relation between proliferation and shape change in the face. (A) Workflow to relate the shape and proliferation atlases of the previous embryonic age N-1 to the shape of each of the samples belonging to the present embryonic age N. All embryos were subject to Procrustes superimposition to remove size difference (not shown). A differential volume (-1,0,+1) for each sample at age N is obtained by comparing to the mesenchyme of the age N-1 atlas (middle row). Then, each voxel of the differential volume at age N was associated with the corresponding voxel of the proliferation atlas of age N-1. Finally, Pearson's linear correlation coefficient is computed only in a mask corresponding to the mesenchyme of the face. (B) Following size removal, box-plots showing the proportion of voxels in the fixed mask of the face that presents outward (+1) or inward (-1) shape change between the consecutive ages studied in this work (E10.0-E11.5).

4) The statement "…our findings do not support the view that spatial patterning of proliferation accounts for differences in morphology among individuals at a given stage" is misleading. This study is vastly underpowered to detect such small differences, so the findings neither support nor refute such a statement. The authors should remove this paragraph or, if they wish do discuss individual variation, they should simply state that much larger studies are necessary to determine the association of proliferation and individual differences.

We have removed this paragraph, however, by using our method to detect a difference between phenotypically similar mutant lines and detecting a proliferation difference between genotypes we show that this method is capable of such studies and further work will be needed to determine the association of proliferation and individual differences.

(Suppl Figure Apoptosis)

Response to concern: (Currently line 177 in source code)
Minor issues:

Line 921: Should this read: the relationship "between" the 3D Volumetric patterning…
Solved

Line 923: "two block two-block…" should this just be two-block? Solved

Response to concern: We thank the reviewer for pointing out these grammatical errors in the article. We have corrected them.

***** Reviewer 3 Advance Summary and Potential Significance to Field:
This methods paper tries to tackle one of the holy grails of developmental biology i.e., relating the morphological changes observed in development to the cellular processes that drive/generate them. It uses the emerging lightsheet microscopy (LSM) to obtain cellular level resolution on whole embryo level. At analytical level it leverages another emerging analytical methodology, deep-learning to effective segment cells and tissues. It has the potential to be impactful in the field of morphogenesis and early development, and when executed right allow to investigate cellular mechanisms that result in developmental defects and dysmorphologies in a direct manner.

Reviewer 3 Comments for the Author:
I have couple significant issues with the manuscript.

1. For methods and technique paper, I do not think the paper stands by itself. THere is extensive reference to other published articles (e.g., Unet model for cellular segmentation). I would have preferred to see more technical implementation detail without having to read the

© 2025. Published by The Company of Biologists under the terms of the Creative Commons Attribution License (https://creativecommons.org/licenses/by/4.0/).

previous manuscripts. I would encourage the authors to shorten the really extensive introduction section, which goes on quite a bit of shortcomings of prior imaging modalities (such as microCT and OPT), and actually expand the methods section.

Response to concern: We thank the reviewer for suggesting ways to improve the writing of this article as a methods article. We have worked on improving the Methods section, which additions and corrections are detailed below.

Action taken:

In the Methods section, page …, line …. we changed the sentences:
"The U-net for tissue segmentation was trained using Nuclear Green images (4096x4096 downsized to 256x256), 21 images from E11.0 embryos and 10 images from E11.5 embryos. For a test set of 8 images from a E11.0 embryo and 10 from a E11.5 embryo, the U-net reached an accuracy of 0.806 and a Dice score of 0.727."
To:
"An U-net for tissue segmentation (Supplementary Material Fig. …) was trained using Nuclear Green images (4096x4096 downsized to 256x256), 21 images from E11.0 embryos and 10 images from E11.5 embryos, annotated by two expert observers. The U-net was trained using an equally weighted combined loss function of soft-Dice and categorical cross-entropy, RMSprop optimizer with learning rate 0.001, for 100 epochs. Training data augmentation based on deformations (flipping, affine deformations) and based on intensities (blurring, brightness, contrast) was performed on the fly (A. B. Jung. 2020). For a test set of 8 images from a E11.0 embryo and 10 from a E11.5 embryo, annotated by a third observer, the U-net reached an accuracy of 0.806 and a Dice score of 0.727."

Supplementary Material Fig. 1:

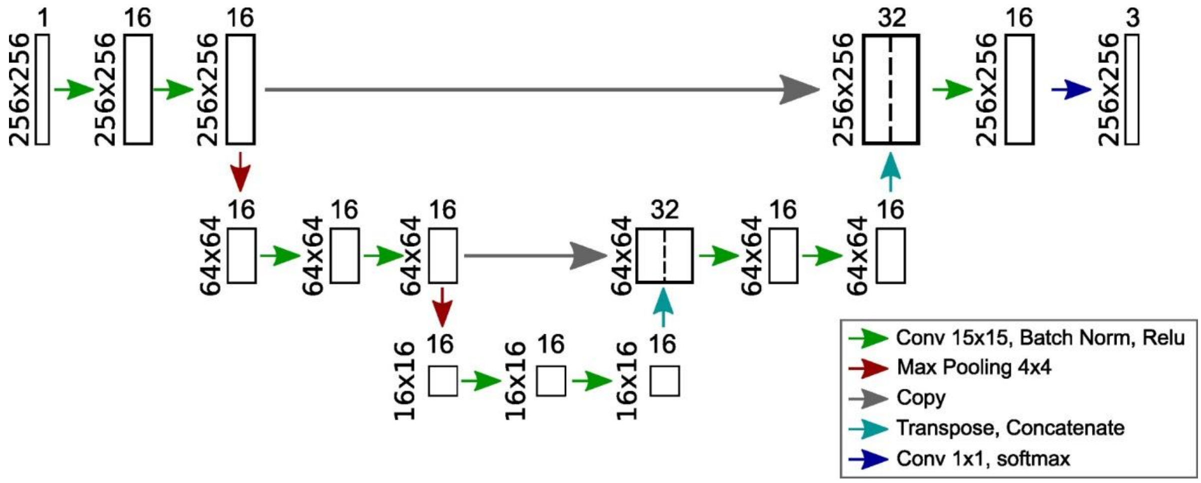

Supplementary Figure 1 : U-net architecture for tissue segmentation. Implemented in TensorFlow-Keras. It was trained using an equally weighted combined loss function of soft-Dice and categorical cross-entropy, RMSprop optimizer with learning rate 0.001, for 100 epochs. Training data augmentation based on deformations (flipping, affine deformations) and based on intensities (blurring, brightness, contrast) was performed on the fly (A. B. Jung. 2020. Imgaug).

In the Methods section, page …, line …. we changed the sentences:
"Then, a landmark-based rigid registration was performed between each of the remaining samples

© 2025. Published by The Company of Biologists under the terms of the Creative Commons Attribution License (https://creativecommons.org/licenses/by/4.0/).

in the age group and their reference sample, using five landmarks placed in the head by an expert observer (Devine et al., 2022) via SimpleITK in Python (Lowekamp et al., 2013)). Then, the resulting transformation was applied to the correspondent tissue segmentation. A groupwise-affine registration was performed among the ten registered tissue segmentations using SimpleElastix in Python (multiresolution registration with 5 number of resolutions, 30000 maximum iterations, linear interpolator, nearest neighbor interpolator for resampling) (Marstal et al., 2016; Devine et al., 2020)."
To
"Then, a landmark-based rigid registration was performed between each of the remaining samples in the age group and their reference sample, using five landmarks placed in the head (Supplementary Figure 2A) by an expert observer (Devine et al., 2022) via SimpleITK in Python (Lowekamp et al., 2013)). Then, the resulting transformation was applied to the correspondent tissue segmentation. A groupwise-affine registration was performed among the ten registered tissue segmentations using SimpleElastix in Python. It was computed using multiresolution registration with 5 number of resolutions, 30000 maximum iterations, linear interpolator, nearest neighbor interpolator for resampling (Supplementary Figure 2B) (Marstal et al., 2016; Devine et al., 2020)."

In the Methods section, page …, line …. we changed the sentences:
"The chain of rigid and affine registrations of each sample was later applied to the corresponding volumes of nuclei and proliferating cells. The mean proliferation per voxel was computed using a 0.15mm×0.15mm×0.15mm window. This proliferation map of the sample is normalized using percentile-based equalization Weigert et al. (2018). The mean proliferation map for each age was obtained by averaging the proliferation maps of the five embryos and their mirrored maps (Fig. 3A)." To:
"The chain of rigid and affine registrations of each sample was later applied to the corresponding volumes of nuclei and proliferating cells (Supplementary Figure 2C,D). The mean proliferation per voxel was computed using a 0.15mm×0.15mm×0.15mm window. This proliferation map of the sample is normalized using percentile-based equalization Weigert et al. (2018) (Supplementary Figure 2E). The mean proliferation map for each age was obtained by averaging the proliferation maps of the five embryos and their mirrored maps (Fig. 3A)."

In the Methods section, page …, line …. we changed the sentences:
"To analyze the shape and proliferation variation among all the samples used in this work, 37 landmarks were placed in the face of the 20 samples and their mirrored volumes by an expert observer (Percival et al., 2014)."
To:
"To analyze the shape and proliferation variation among all the samples used in this work, 37 landmarks were placed in the face of the 20 samples and their mirrored volumes by an expert observer (Supplementary Figure 2B) (Percival et al., 2014)."

Supplementary Material Fig. 2:

© 2025. Published by The Company of Biologists under the terms of the Creative Commons Attribution License (https://creativecommons.org/licenses/by/4.0/).

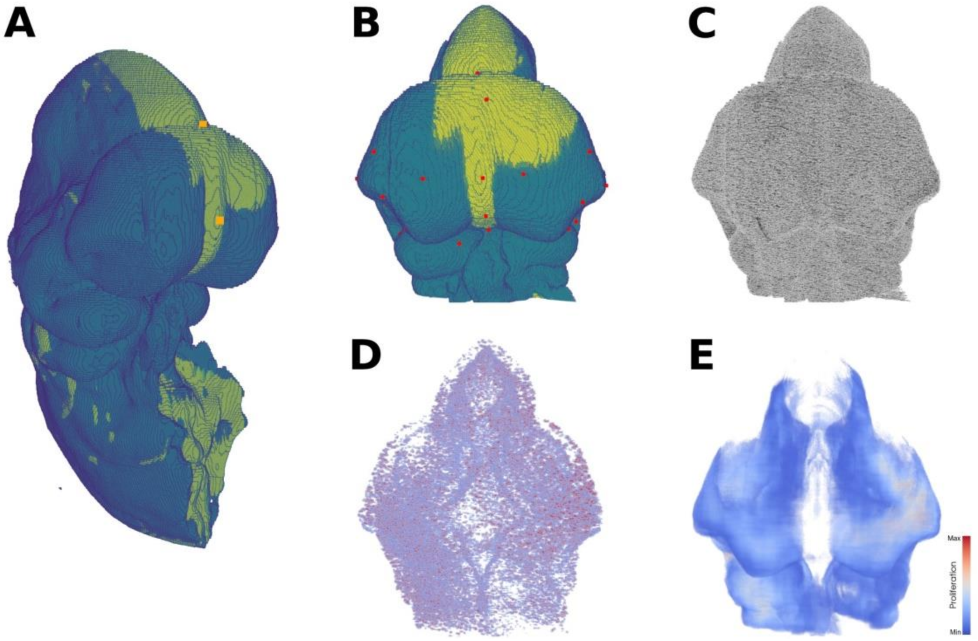

Supplementary Figure 2 : Example of segmentations, transformations and landmark configurations for a E10.0 sample (Dec2_E10_12). (A) Tissue segmentation of the full embryo (mesenchyme in teal, neural ectoderm in yellow) and its five landmarks (orange) for the initial rigid registration to a common reference for atlas construction. (B) Segmentation-based groupwise-affine registration result, for atlas construction. The thirty-seven landmarks used for later bulk analysis are shown in red. (C) Nuclei segmentation isotropic volume after applying the result of the groupwise-affine registration. (D) Proliferating cell segmentation isotropic volume after applying the result of the groupwise-affine registration. (E) Proliferation map in mesenchyme after convolving the volume shown in D and masking with the volume shown in B.

2. I was a little surprised and not entirely clear why the stage atlases were created using only affine registration? Affine transformation is a linear transformation that only accounts for global size differences. Given the rapid pace of development at E9 and E10, I would have expected there are many morphological differences in the embryos (even if they were from timed matings) that will not be sufficiently captured by an affine only atlas building process. I was not able to locate the atlas files to check this for myself. I would strongly encourage the authors to submit their atlases as part of their data repository (and if they did I think it should have been more clear in the manuscript).

Response to concern: We agreed with the reviewer that the decisions about the different transformations needed better explanations. We have incorporated them in the article. At the time of submission of the article, the publicly available data repository was under the publication process and it became publicly available soon after (https://doi.org/10.5683/SP3/AXMCDE). This dataset is composed by the tissue segmentation, proliferating cell segmentation, and landmarking for each sample used in this article, and the tissue and proliferation atlases for each embryonic age. We have added the reference in the article.

Action taken:

In the LSFM as a tool section, page 14, line 1491 we changed the sentences:

"In this work, the more pronounced image artifacts (blurriness, signal loss) in the older embryos (E10.0-E11.5) resulted in poorer performance for the U-nets for tissue segmentation compared to E9.5-E10.5 embryos. This occurred even when U-nets were re-trained with images from our data. As a result, user intervention was required to correct 3D tissue models in cases where

© 2025. Published by The Company of Biologists under the terms of the Creative Commons Attribution License (https://creativecommons.org/licenses/by/4.0/).

tissues were misclassified. Current work is focused on solving or mitigating these issues so that we can apply non-linear registration methods that have less tendency to introduce noise during registration to the atlas."
To:
"In this work, the more pronounced image artifacts (blurriness, signal loss) in the older embryos (E10.0-E11.5) resulted in poorer performance for the U-nets for tissue segmentation compared to E9.5-E10.5 embryos. This occurred even when U-nets were re-trained with images from our current data, using a larger patch size to compensate for larger embryo size than previous work and to augment tolerance to artifacts. As a result, user intervention was required to correct 3D tissue models in cases where tissues were misclassified. These segmentation errors, in association with the limited sample size and high shape variation during early mouse development limited the degrees of freedom of the registration at the time of building atlases, to restrict the introduction of noise to them (i.e. developmental exceptions in samples, unreal structures or processes due to imaging artifacts, among others). Current work is focused on solving or mitigating these issues so that we can apply non-linear registration methods to the atlas construction, to produce atlases with a higher level of detail while keeping certainty."

3. On Figure 5 the colorbar does not indicate the magnitude of scale. I imagine red are higher magnitude proliferation and blue is lower, but please do not make us guess. Also what is the unit? In general I find the yellow/green color rendering very difficult to follow. Also this being 3D data (and more importantly analysis), I can't interpret what's going on inside the embryo. I think if the authors can provide the actual 3D models (or better yet voxel maps) in an open-source format (since they are already using 3D Slicer for part of their workflow) it will be better than a static 2D image.

Response to concern: We thank the reviewer for noticing the missing labels in the proliferation scale bar. The green and yellow colouring of the atlases is simply a label to differentiate mesenchymal tissue (used in the analysis) from neural ectoderm (left out). The colour maps were selected to be colour-blind friendly, consistent though figures, and to maximize their difference to avoid confusions, factors that limited the available colour maps for an article with many different maps. In line with this suggestion of the reviewer, the volumes are available in our repository in multipage TIFF format that can be opened with Paraview or 3D Slicer for 3D visualization, and 2D image viewers such as XnView (https://doi.org/10.5683/SP3/AXMCDE).

Action taken:
Clarification has been added to the figure legend stating the colour meanings in each part of the figure. The proliferation scale used is the same throughout Figures 3-5. We have added clarification to the legend of Figure 2, where it first is used, regarding the magnitude of scale: "(minimum proliferation in blue, maximum proliferation in red)"
In Figure 5, we clarified the caption: "A differential volume (-1,0,+1) for each sample at age N is obtained by comparing to the mesenchyme of the age N-1 atlas (middle row, label -1 in purple, +1 in gold)."

We changed the Data availability section from: "Insert the Data availability text here."
TO:
"The source code produced and used in this work can be found in https://github.com/lucaslovercio/LSMprocessing. Data can be found in https://doi.org/10.5683/SP3/AXMCDE."

4. In some ways it is not surprising to have high correlation between higher proliferation and morphological changes, but it is of course it is still a nice validation. What was surprising to me is the lack of correlation between apoptosis and morphological change. Because it is essentially the flip side of the same argument made by the authors in L1064. It wasn't clear this was not investigated or it was investigated and found not to be significant and hence not reported.

© 2025. Published by The Company of Biologists under the terms of the Creative Commons Attribution License (https://creativecommons.org/licenses/by/4.0/).

In our early data, we stained for both proliferation and apoptosis. We observed very little apoptosis before E11.5-E12.0, when the arch artery begins to regress. Outside of the arch artery regression and a burst of apoptosis during fusion of the nasal processes, sporadic apoptosis was low and uniform, and therefore doesn't appear to have a major role in aligning the facial processes during these early events. As such, we excluded apoptosis from further analyses. We have added a supplementary figure including apoptosis images and an accompanying sentence in the body clarifying this.

Second decision letter

MS ID#: dev.204511

MS TITLE: Quantifying the relationship between cell proliferation and morphology during development of the face

AUTHORS: Rebecca M Green, Lucas Lo Vercio, Andreas Dauter, Elizabeth C Barretto, Jay Devine, Marta Vidal Garcia, Marta Marchini, Samuel Robertson, Xiang zhao, Anandita Mahika, M Bilal Shakir, Sienna Guo, Julia C Boughner, Heather Szabo-Rogers, Wendy Dean, Arthur D. Lander, Ralph S. Marcucio, Nils D Forkert, and Benedikt Hallgrimsson

Dear Dr Green,

I have now received all the referees reports on the above manuscript, and have reached a decision. The referees' comments are appended below, or you can access them online: please go to .

The overall evaluation is positive and we would like to publish a revised manuscript in Development, provided that the referees' comments can be satisfactorily addressed. Please attend to all of the reviewers' comments in your revised manuscript and detail them in your point-by-point response. If you do not agree with any of their criticisms or suggestions explain clearly why this is so. If it would be helpful, you are welcome to contact us to discuss your revision in greater detail. Please send us a point-by-point response indicating your plans for addressing the referees' comments, and we will look over this and provide further guidance.

SUMMARY OF THE ADVANCE MADE IN THIS PAPER AND ITS POTENTIAL SIGNIFICANCE TO THE FIELD

The authors have responded directly, comprehensively, and satisfactorily to all points in my review. I am now happy to recommend acceptance for publication in Development.

SUGGESTIONS TO AUTHORS

Minor concerns:

Figure 8: panel A and B should be labeled with their respective genotypes. The type of staining performed should be indicated. In addition, Lantz et al. in 2020 have reported that Unicorn embryos have decreased proliferation in caudal MNPs by using BrdU and pHH3 staining on sections of mutants vs controls; can the authors elaborate on their findings and report if/how differences in proliferation are observed when they analyze these mutant embryos using their pipeline? This would allow for a direct comparison between the new methodology described here and the classical methods used to measure proliferation.

SUMMARY OF THE ADVANCE MADE IN THIS PAPER AND ITS POTENTIAL SIGNIFICANCE TO THE FIELD

© 2025. Published by The Company of Biologists under the terms of the Creative Commons Attribution License (https://creativecommons.org/licenses/by/4.0/).

In this manuscript Lo Vercio and colleagues use light sheet microscopy coupled with machine learning-based tools to study how proliferation affects 3D morphology during facial growth of mouse embryos. The pipeline developed by the authors includes automated segmentation followed by landmark-based morphometric analyses and quantification of cell proliferation. By applying this new methodology, the authors conclude that even though no strong differences in proliferation density were observed within the growing prominences, there is a statistically significant correlation between proliferation and shape change. Of note, in the revised manuscript the authors have conducted additional experiments and used their pipeline to test the role of proliferation in facial morphology in a mutant mouse model, the "Unicorn" ENU mutant, which has a subtle midfacial phenotype.
Limitations of the study:
- Difficulties in segmenting neural ectoderm does not allow to explore potential different roles of epithelial vs mesenchymal cell proliferation.
- The pipeline does not take into account the contribution of other cellular behaviors such as cell migration or cell apoptosis.
Importantly, these limitations have been discussed in the paper.
Overall, the data presented in the manuscript are solid and convincing. This approach advances the fields of morphogenesis and early development, providing a new tool to quantify cellular mechanisms contribution to morphogenic changes.
For these reasons this reviewer believes that the manuscript meets the criteria for publication in Development.

Second revision

Author response to reviewers' comments

We want to thank the editors and reviewers for their time on this manuscript. We have made the changes suggested by reviewer 2 and feel that they strengthen the manuscript.

Comments from the Reviewers:
Reviewer 1: SUMMARY OF THE ADVANCE MADE IN THIS PAPER AND ITS POTENTIAL SIGNIFICANCE TO THE FIELD

The authors have responded directly, comprehensively, and satisfactorily to all points in my review. I am now happy to recommend acceptance for publication in Development.

Reviewer 1: Thank you to the reviewer.

SUGGESTIONS TO AUTHORS
Minor concerns:
Figure 8: panel A and B should be labeled with their respective genotypes. The type of staining performed should be indicated. In addition, Lantz et al. in 2020 have reported that Unicorn embryos have decreased proliferation in caudal MNPs by using BrdU and pHH3 staining on sections of mutants vs controls; can the authors elaborate on their findings and report if/how differences in proliferation are observed when they analyze these mutant embryos using their pipeline? This would allow for a direct comparison between the new methodology described here and the classical methods used to measure proliferation.

Reviewer 2: Thank you for your comments, this paper is not without limitations and we are glad you feel that they were adequately addressed.

We have addressed your other concerns in both the results and conclusions sections.

© 2025. Published by The Company of Biologists under the terms of the Creative Commons Attribution License (https://creativecommons.org/licenses/by/4.0/).

In the results section, we added additional labels to figure 8 panel A and B. In order to better illustrate the differences between the groups of embryos, we have transformed the PC3 results into a visual representation of the proliferation along that axis, added these to the figure as panel D and added the following text to the result section. " Further analysis of the proliferation patterns associated with the PC3 shows overall decreased proliferation in the whole nasal capsule and maxilla, with the strongest changes in the lateral nasal process. While these images do not show an exact proliferation pattern for each embryo, they give an overall idea of the patterns of change."

In the discussion section we have added the following text:
The paper describing the Unicorn mice observed decreased proliferation primarily in the cadual edge of the medial nasal process mesenchyme. They did not examine the lateral edge (Lantz). While it is difficult to directly compare spot methods to our method, we observe more widespread differences. This was unsurprising as the shape changes are throughout the nasal capsule, therefore it is logical that there are more proliferation differences than were captured in the original study.

While there are limitations with direct comparisons, we hope that this visual and additional discussion helps strengthen the utility of this method.

Thank you for your consideration of this resubmission.

Third decision letter

MS ID#: dev.204511R1

MS TITLE: Quantifying the relationship between cell proliferation and morphology during development of the face

AUTHORS: Rebecca M Green, Lucas Lo Vercio, Andreas Dauter, Elizabeth C Barretto, Jay Devine, Marta Vidal Garcia, Marta Marchini, Samuel Robertson, Xiang zhao, Anandita Mahika, M Bilal Shakir, Sienna Guo, Julia C Boughner, Heather Szabo-Rogers, Wendy Dean, Arthur D. Lander, Ralph S. Marcucio, Nils D Forkert, and Benedikt Hallgrimsson

Dear Dr Green,
I am happy to tell you that your manuscript has been accepted for publication in Development, pending our standard publication integrity checks.

© 2025. Published by The Company of Biologists under the terms of the Creative Commons Attribution License (https://creativecommons.org/licenses/by/4.0/).
